# Changes in microbial community succession and volatile compounds during the natural fermentation of bangcai

**DOI:** 10.3389/fmicb.2025.1581378

**Published:** 2025-04-02

**Authors:** Lili Xie, Xueli Wang, Xiujun Wang, Xueting Liu

**Affiliations:** ^1^Province Key Laboratory of Fermentation Engineering and Biological Pharmacy, Guizhou University, Guiyang, China; ^2^College of Liquor and Food Engineering, Guizhou University, Guiyang, China

**Keywords:** *Brassica juncea var. crassicaulis*, natural fermentation, metagenomics, microbial community, volatile compounds, correlation analysis

## Abstract

**Introduction:**

Fermented bangcai (*Brassica juncea* var. *crassicaulis*) is a traditional Chinese food with unique flavor. However, the formation mechanism of flavor compounds related to the fermentation process of bangcai has not been thoroughly studied.

**Methods:**

Gas chromatography-ion mobility spectrometry technology combined with metagenomics was used to analyze the characteristic volatile flavor compounds and microbial community structure of bangcai before and after fermentation in this study.

**Results:**

A total of 91 types of volatile organic compounds were detected in this study. The pungent odor brought by allyl isothiocyanate, 1-butene isothiocyanate, and other substances in the raw materials was removed through fermentation. This process led to the formation of flavor substances such as propyl acetate, ethyl acetate, and 2-methyl-3-furanthiol, which imparted bangcai with flavors of flower and fruit, roast meat, and fried coffee. In addition, our study found that after air drying, bangcai mainly contained γ-butyrolactone, nonanal and other flavor compounds, giving the bangcai products a richer floral and fruity flavor profile. *Citrobacter*, *Lactobacillus*, and *Leuconostoc* were the dominant bacteria in the fermentation process of bangcai. They were significantly related to the formation of differential flavor compounds such as γ-butyrolactone, ethyl 2-methylpropanoat, and benzaldehyde-*D*.

**Discussion:**

These results provide a theoretical basis for improving the flavor quality of fermented vegetable products.

## 1 Introduction

*Brassica juncea* var. *crassicaulis* (bangcai) is an annual herbaceous plant belonging to the *Brassicaceae* family, which is widely distributed in the southwestern regions of China and the Yangtze River basin (Liu et al., 2017). After undergoing traditional fermentation processing, its stems form unique flavored products. This process not only effectively degrades the pungent isothiocyanate compounds (such as allyl isothiocyanate) in bangcai, but also generates a diverse array of volatile organic compounds (VOCs) through microbial metabolism, imparting characteristic sensory qualities to the fermented products (Xiong et al., 2024). Research has shown that VOCs, such as heptanal, nonanal, and 2-pentylfuran, produced during the fermentation of bangcai, exhibit significant dynamic variations, with their formation closely related to the succession of the microbial community (Wang et al., 2024). Among these, lactic acid bacteria (LAB), such as *Lactobacillus*, *Leuconostoc*, and *Weissella*, serve as the dominant functional microbiota in the fermentation system. By regulating pathways such as acetate metabolism, citrate conversion, and heterolactic fermentation (Indrati et al., 2022; Rocchetti et al., 2019), LAB drive the synthesis of flavor precursor compounds, like organic acids and free amino acids, and further catalyze the production of characteristic flavor components, including alcohols, esters, and sulfides (Yu et al., 2022b).

Traditional studies have frequently used 16S rRNA gene sequencing combined with gas chromatography-mass spectrometry (GC-MS) to analyze the correlation between microbial communities and VOCs. A study by Yu et al. (2022b) found a positive correlation between LAB and the relative contents of ethyl acetate and butyric acid during the fermentation of mustard [*Brassica juncea* (L.)] greens. Similarly, Chen et al. (2023) also discovered that the key VOCs in fermented pickled ginger products, such as myrcene, geranyl acetate, citral, and geranyl, showed a certain correlation with the bacterial genera in the fermentation process. However, 16S rRNA sequencing technology is limited by its resolution, which typically only reaches the genus level, and its inability to provide functional annotations. This limitation complicates the precise analysis of the microbial metabolic networks and the molecular mechanisms of VOC biosynthesis, severely hindering the development of flavor regulation strategies for fermented vegetables.

In recent years, metagenomics technology has been widely applied to study fermented vegetables (Park et al., 2019). Compared with 16S rRNA high-throughput sequencing, metagenomics technology can not only fully explore the microbial genome information of samples but also study the functions and metabolic pathways of microorganisms (Turnbaugh and Gordon, 2008). Das and Tamang (2023) analyzed the microbial community structure of Indian palm wine using metagenomics technology and predicted the biosynthetic pathways of ethanol, acetic acid, butyric acid, linalool, and riboflavin using the KEGG/COG database. Based on the combined analysis of metagenomics and metabonomics, Wang et al. (2020) found that *Halanaerobium*, *Halomonas*, *Tetragenococcus*, *Halococcus*, and *Candidatus Frackibacter* were the dominant bacteria in the process of fish sauce fermentation. These bacteria were closely related to the synthesis of flavor compounds. On this basis, the metabolic pathway network of volatile flavor compounds was constructed, and the formation mechanism of characteristic flavor compounds was explored. In conclusion, metagenomics can more comprehensively characterize the composition of complex microbial communities in fermented food and complete the metabolic and functional prediction analysis of microbial communities.

Therefore, this study used metagenomics technology to analyze the microbial community structure and function during the natural fermentation process of bangcai. In addition, this study combined gas chromatography-ion mobility spectrometry (GC-IMS) technology to compare the composition and content changes in VOC before and after bangcai fermentation. A symbiotic network between microorganisms and differential VOCs was constructed to analyze and elucidate their potential relationship. The results of this study provide a theoretical basis for improving the fermentation process of bangcai and enhancing the flavor quality of the final product.

## 2 Materials and methods

### 2.1 Sample collection and preparation

**Figure 1:**
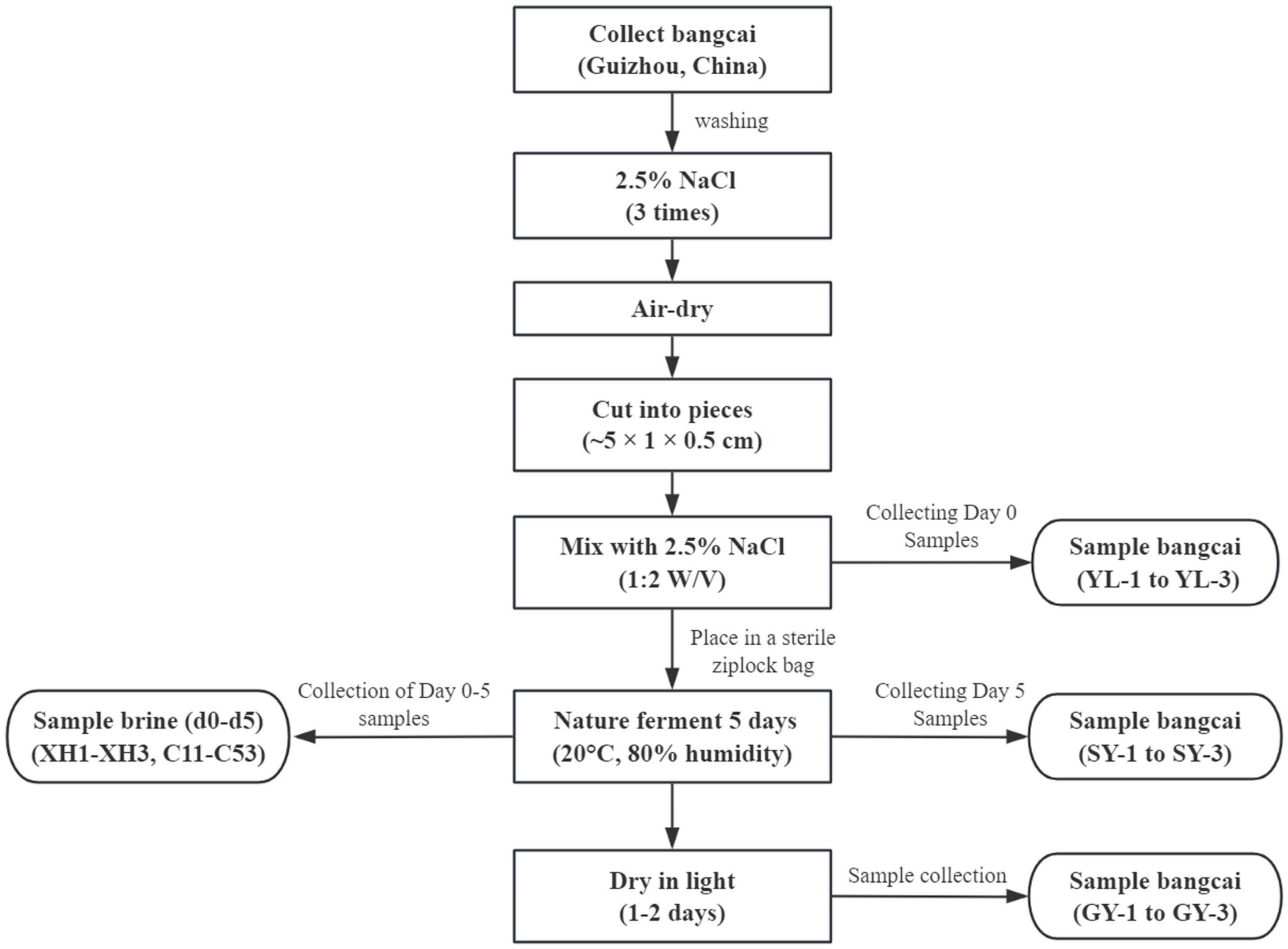
Flowchart showing the processing steps and sampling stages of bangcai during natural fermentation.

As shown in Figure 1, fresh bangcai (*Brassica juncea* var. *crassicaulis*) was collected from Li’s Food Co., Ltd. (Zhenyuan County, Qiandongnan Prefecture, Guizhou Province, China). After collection, the fresh bangcai was thoroughly washed three times with a 2.5% sterile sodium chloride solution, then air-dried. Next, the dried bangcai stems and stalks were cut into pieces measuring approximately 5 cm × 1 cm × 0.5 cm and added with a 2.5% sodium chloride solution at a ratio of 1:2 (W/V). Then, the mixture was placed into a sterile sealed bag. Finally, the product was placed in a factory for natural fermentation for 5 days. The fermentation temperature was 20°C ± 2°C, and the humidity was approximately 80%. After fermentation, the product was exposed to natural light for 1–2 days to complete the drying process.

During the fermentation process of bangcai, fermentation broth samples were randomly collected on days 0–5 (d0, d1, d2, d3, d4, d5) for the extraction and analysis of total DNA. Each time point was sampled in triplicate. Samples collected on day 0 were labeled XH1–XH3, and those collected on days 1–5 were labeled C11–C53. For the convenience of subsequent analysis, the collected fermentation broth samples were divided into six groups, corresponding to the samples collected on days 0–5 (d0, d1, d2, d3, d4, d5) respectively. Additionally, raw bangcai samples (labeled YL-1 to YL-3), wet bangcai samples containing salt water collected on the fifth day of fermentation (labeled SY-1 to SY-3) and dried bangcai samples after drying (labeled GY-1 to GY-3) were collected. The collected samples were mainly used to determine volatile flavor compounds.

### 2.2 Analysis of volatile compounds

The types and relative contents of VOC during the fermentation process of bangcai were determined using GC-IMS (FlavourSpec^®^, Germany), following the method described by Liu et al. (2021). The specific procedure was as follows: initially, 2.0 g of crushed and homogenized bangcai samples were accurately weighed into a 20 mL headspace vial. Next, 6 mL of 95°C ultrapure water was added to the headspace vial and mixed for 10 min. After cooling to room temperature, the samples were placed in a 60°C, 500 r/min condition for 15 min of incubation before analysis, with an injection volume of 500 μL.

The conditions for GC-IMS were as follows: carrier gas, N_2_ (purity ≥99.999%); injection temperature, 85°C; MXT-5 chromatographic column, 15 m × 0.53 mm, 1 μm (RESTEK, United States); column temperature, 60°C; and carrier gas flow rate, 2 mL/min for 0–2 min and 100 mL/min for 2–20 min.

The IMS conditions were as follows: drift tube length, 98 mm; drift tube temperature, 45°C; drift gas, N_2_ (purity ≥99.999%); drift gas flow rate, 150 mL/min; beta radiation, with 3H as the radioactive source; and positive ionization mode. The content of volatile flavor components is expressed as the normalized relative peak area (%).

### 2.3 Genomic DNA extraction and metagenomic sequencing

Genomic DNA extraction and metagenomic sequencing were carried out following the method described by Yu et al. (2022a), with modifications. The fermentation broth samples collected in section 2.1 were centrifuged at 12,000 rpm for 10 min using a centrifuge (D1542R Desktop Centrifuge, DLAB, China). The sediment was collected in a 2 mL EP tube, and 1,000 μL of CTAB lysis buffer and lysozyme were added. The EP tube was then placed in a 65°C water bath for 1 h and inverted several times every 15 min to ensure complete lysis of the sample. Subsequently, 1,000 μL of a mixed solution (pH 8.0, phenol/chloroform/isoamyl alcohol in a 25:24:1 ratio) was added to the cracked sample, which was then inverted, thoroughly mixed, and centrifuged at 12,000 rpm for 10 min. The supernatant was collected and mixed with an equal volume of a mixture of chloroform and isoamyl alcohol (volume ratio, 24:1). The sample was then inverted and centrifuged for 10 min under the same conditions. The supernatant was transferred into a 1.5 mL centrifuge tube, added with 0.6 times the volume of isopropanol, shaken up and down, and stored at −20°C for at least 30 min. After precipitation, the sample was centrifuged at 12,000 rpm for 10 min. The supernatant was then poured out, and the precipitate was washed with 1 mL of 75% ethanol. Finally, the remaining liquid was removed by suction. ddH_2_O was successively added to the sediment, followed by 1 μL of RNaseA, and the mixture was allowed to stand at 37°C for 15 min. The Qubit Fluorometric Quantification Kit (Qubit 2.0, Life Technologies, CA, United States) was used to accurately quantify the DNA concentration. The purity and integrity of DNA were analyzed through agarose gel electrophoresis (DYCP-32C Agarose Horizontal Electrophoresis instrument, Beijing Liuyi Instrument Factory, China).

The NEB Next^®^ Ultra^™^ DNA Library Prep Kit from Illumina (NEB, United States) was used to construct the library. The qualified DNA samples were randomly fragmented into 350 bp fragments using an ultrasonic crusher (Covaris M220, Covaris S2 System, MA, United States). Subsequently, the whole library was prepared by repairing the terminal, adding a tail, incorporating a sequencing connector, and conducting purification, PCR amplification (T100PCR, United States), and other steps. Finally, the PCR product was purified using the AMPure XP system. The insert size of the library was detected using an Agilent 2100 biological analyzer (Agilent 2100, Agilent Technologies Co. Ltd., United States), and the concentration of the library was analyzed quantitatively by real-time PCR.

After the library was constructed, Qubit 2.0 was used for preliminary quantification, and the library was diluted to 1 ng/μL. The Agilent 2,100 was used to detect the inserted fragments of the library. After the inserted fragments reached the expected size, the effective concentration of the library was accurately quantified by qPCR. Then, after the library passed the test, the different libraries were pooled into a flow cell according to the required effective concentration and target offline data volume. After clustering, the samples were sequenced using the Illumina PE150 (2 × 150) high-throughput sequencing platform (NovaSeq 6000, Illumina, San Diego, CA, United States).

### 2.4 Metagenomic bioinformatic analyses

#### 2.4.1 Data quality control and dehosting sequence

Using the Illumina NovaSeq high-throughput sequencing platform for metagenome sequencing, raw data of bacteria, fungi, and viruses in bangcai fermentation broth samples was obtained.

To ensure the reliability of data, KneadData software was used to preprocess raw sequencing data. The specific processing steps were as follows: (1) The connector sequence (based on Trimmomatic, parameter:illuminaclip:adapters_path:2:30:10) and the sequence with low quality (default quality score threshold ≤20) (based on Trimmomatic, parameter: slidingwindow:4:20) were removed. In addition, the sequence with a final length less than 50 bp (based on Trimmomatic, parameter:minlen:50) was removed. (2) Considering that the sample might be contaminated by the host, it was necessary to compare the clean data with the host genome. By default, Bowtie2 software^1^ (with very sensitive parameters) was used to filter the sequences from the host and obtain valid sequences for subsequent analysis. (3) Finally, the rationality and effect of quality control were tested using fastqc (Langmead and Salzberg, 2012).

#### 2.4.2 Species notes

Kraken2 and the self-built microbial nucleic acid database (used to screen the sequences of bacteria, fungi, archaea, and viruses in the NCBI NT nucleic acid database and the RefSeq genome database) were used to calculate the number of species found in the sample, and Bracken was used to predict the actual relative abundance of species in the sample. Kraken2 is the latest comparison software based on K-mer. The local Kraken2 database we used contains 16,799 known bacterial genomes (Brum et al., 2015).

#### 2.4.3 Comments based on the reads function

Using HUMAnN2 software, the sequences, after quality control and host removal, were compared with the protein database (UniRef90) (based on DIAMOND). Moreover, the annotation information and a relative abundance table for each functional database were obtained according to the correspondence between the UniRef90 ID and each database (Segata et al., 2011).

Based on the species and the functional abundance tables, abundance clustering analysis can be performed. Using grouping information, LEfSe biomarker analysis was conducted to explore the differences in species and functional compositions between samples (Villar et al., 2015).

### 2.5 Statistical analyses

One-way analysis of variance was conducted using SPSS statistical software version 25.0 (IBM, Armonk, NY, United States), with significance determined at *p* < 0.05. *Post hoc* tests were performed using Tukey’s method. Data were organized and summarized using Excel, and plots were generated using the online platform Sangerbox. GC-IMS data were analyzed using the instrument’s proprietary software, Laboratory Analytical Viewer, and Library Search Software (with built-in NIST2014 and IMS databases) was used for matching and analysis of detected VOCs. Metabolomics analysis tools^2^ were used for principal component analysis (PCA) and orthogonal partial least squares-discriminant analysis (OPLS-DA) of the VOCs. Spearman’s rank correlation was employed to assess the correlation between microbial abundance and volatile flavor compounds.

## 3 Results and discussion

### 3.1 Analysis of volatile metabolites

#### 3.1.1 Overview of volatile metabolites

**Figure 2:**
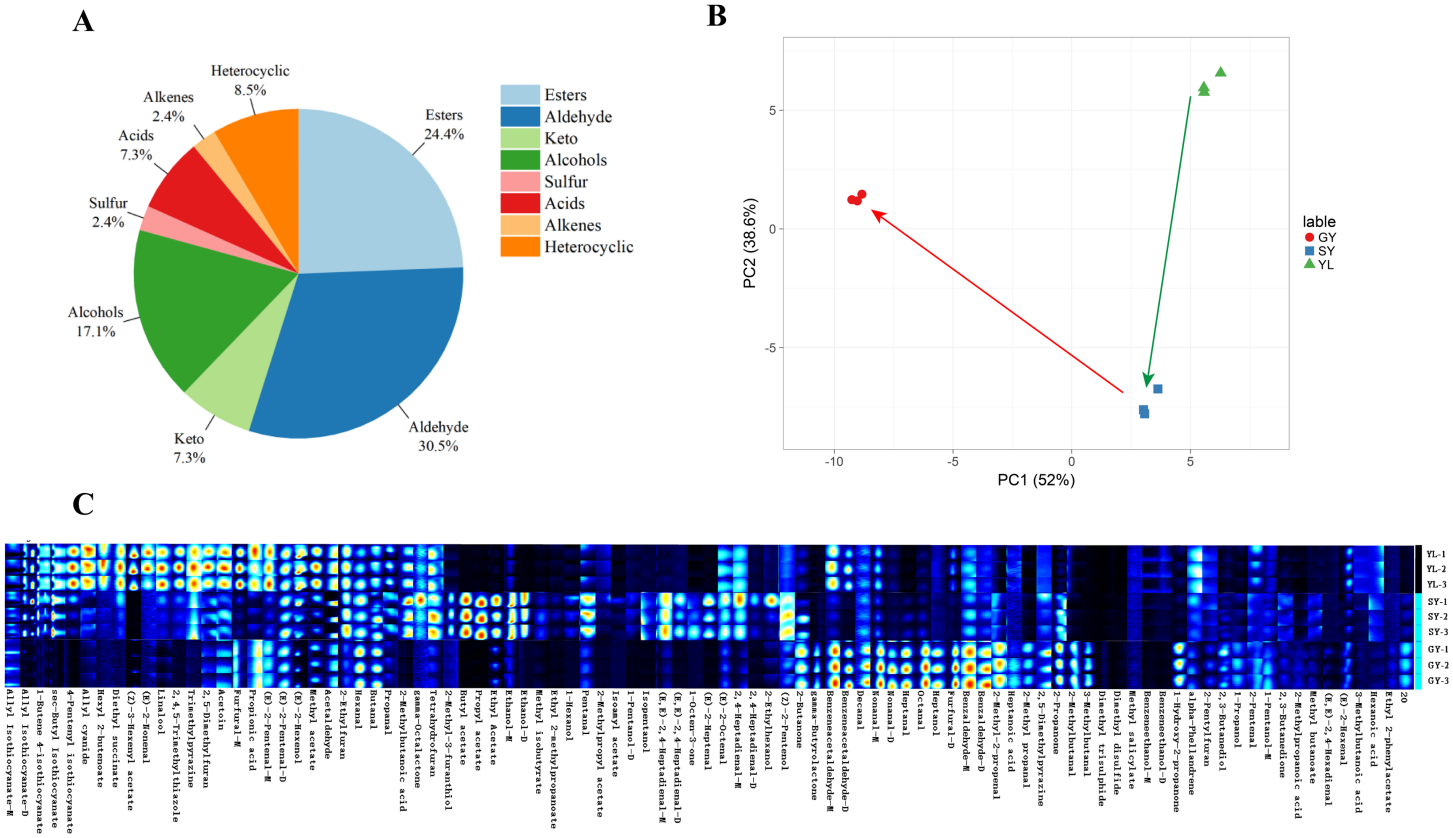
Classification proportion of VOC in the measured samples **(A)**. Principal component analysis of VOC in raw materials and wet and dry samples after the fermentation of bangcai (green and red arrows represent the changes in VOCs in bangcai during fermentation and drying, respectively) **(B)**. Cross flow ion mobility spectrometry (GC-IMS) fingerprints of volatile components in YL, SY, and GY **(C)**. SY: wet sample after 5 days of fermentation; GY: dry sample after natural air drying; YL: bangcai raw material.

Using GC-IMS technology, a total of 91 VOC were detected in the raw material bangcai and the wet and dry samples after fermentation, and the results were shown in Supplementary Tables S1, S2. Based on the chemical structure, all VOC can be divided into eight categories. As can be seen in Figure 2A, aldehydes (30.5%) and esters (24.4%) accounted for more than 50.0% of the total VOCs, followed by alcohols (17.1%), heterocycles (8.50%), acids (7.30%), ketones (7.30%), sulfur (2.40%), and alkenes (2.40%). Our research results show that the composition of VOCs in naturally fermented bangcai is somewhat similar to the study by Jiang et al. (2023) on naturally fermented mustard greens. However, in their study, heterocyclic compounds accounted for the largest proportion, followed by esters and hydrocarbons, which differs from our findings. We speculate that this difference may be due to variations in the raw materials and associated microorganisms. The relative concentration of VOC was analyzed using PCA. As illustrated in Figure 2B, the results revealed a significant difference in the component content among YL, SY, and GY. The total variance of the two principal components could reach 90.6%, indicating a significant difference in the composition of VOCs before and after processing. However, the direction of the arrow also reveals the changing trend of the main components of the flavor compounds in the samples after fermentation and drying. After 5 days of fermentation, the VOCs in the wet sample SY primarily changed in PC1, whereas the VOCs in the GY primarily changed in PC2.

Fingerprints can visually characterize the dynamic changes in flavor compounds among different samples and reflect the complete information of VOC in each sample, as well as the differences in VOC between samples. According to the GC-IMS data, corresponding characteristic fingerprints were established for different samples. Each row in Figure 2C represents all signal peaks selected from a sample, whereas each column represents signal peaks of the same VOCs in different samples. “*_M*” and “*_D*” following certain substances represent monomers and dimers of the same substance, respectively. The color represents the content of VOCs. The brighter the color, the higher the concentration. The VOC of the processed samples (GY and SY) were significantly different from those in the YL samples. The contents of allyl isothiocyanate-*D*, 1-butene 4-isothiocyanate, sec-butyl isothiocyanate, 4-pentenyl isothiocyanate, hexyl 2-butenoate, and other esters in the YL samples were higher. Relevant studies have reported that isothiocyanate has a pungent odor and is representative of glucosinolate hydrolysis in cruciferous vegetables (Cho et al., 2016). In this study, the contents of 2-methyl-3-furanthiol, butyl acetate, propyl acetate, ethyl acetate, methyl butanoate, (*E*,*E*)-2,4-heptadienal-*D*, 2,4-heptadienal, (*E*)-2-heptenal, and other compounds in the SY samples increased after fermentation. 2-Methyl-3-furanthiol contributed roasted meat and fried coffee flavors to the product, whereas butyl acetate, propyl acetate, ethyl acetate, and other esters provided fruit flavors. The sample exhibited a complex flavor overall. After air drying, the GY sample exhibited high contents of γ-butyrolactone, 2-propanone, nonanal-*M*, methyl acetate, (*E*)-2-hexenol, benzeneacetaldehyde-*M*, furfural-*D*, and other compounds. They contributed to the formation of floral aromas, such as apple, rose, and citrus, in the samples (Li et al., 2016).

#### 3.1.2 Volatile compound variance analysis

**Figure 3:**
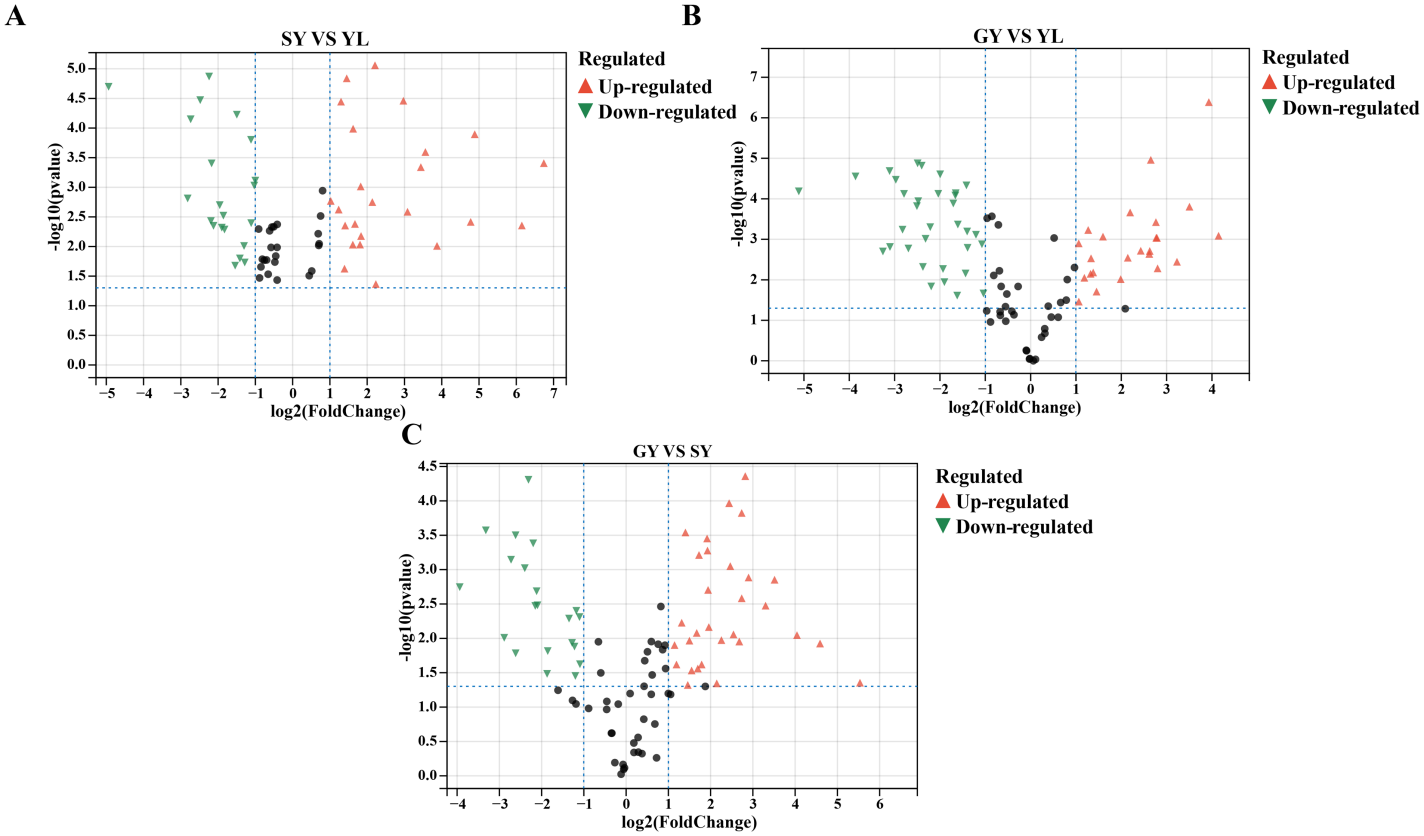
Volcano plots of differentially volatile metabolites in each group **(A–C)** (log_2_ (FC)| >1, *p* < 0.05, and VIP >1). Red triangles represent upregulated metabolites, green triangles represent downregulated metabolites, and black dots represent metabolites with no significant differences.

The raw material bangcai YL was compared with SY after fermentation and GY after air drying. The metabolites with significant differences between the groups were identified using the constructed OPLS-DA model. According to Supplementary Figure S1, *R*^2^*X* is greater than 0.782, *R*^2^*Y* is greater than 0.998, and *Q*^2^ is greater than 0.991 among all groups. These results indicate the reliability and high predictive capability of the model. The volatile metabolites with significant differences were screened under the conditions of log_2_ (FC) >1, *p* < 0.05, and VIP >1. The distribution of different metabolites in different sample groups was characterized by a volcano plot in Figures 3A–C. Using the raw material of bangcai sample YL as the reference, 45 different volatile metabolites were detected in the reference group YL and the fermented sample SY, with 25 metabolites upregulated and 20 downregulated. In addition, 59 differential volatile metabolites were detected in the YL and GY samples after air drying, with 26 metabolites upregulated and 33 downregulated. Among these, the contents of isothiocyanate compounds, such as 4-pentenyl isothiocyanate, 1-butene 4-isothiocyanate, sec-butyl isothiocyanate, and allyl isothiocyanate-*D*, in the two groups were lower than those in the YL, consistent with the research results of Zhang et al. (2022). The reduction in spicy compounds, like allyl isothiocyanate and 1-butene isothiocyanate, during fermentation can be attributed to the hydrolysis of glucosinolates and subsequent microbial degradation of isothiocyanates. Isothiocyanates are formed through glucosinolate hydrolysis (Tarar and Peng, 2022), and as LAB biomass increases, this hydrolysis becomes more efficient under acidic conditions. Additionally, the relative content of aldehydes such as 2-methylbutyraldehyde, Benzeneacetaldehyde-M, Benzeneacetaldehyde-D and (E)-2-Pentenal-M, which are mainly produced through the microbial metabolic pathways of amino acids, also significantly decreased with the increase in fermentation time. Fermented reduces isothiocyanate levels and promotes the formation of other flavor-enhancing volatile sulfur compounds, such as 2-methyl-3-furanthiol and dimethyl trisulfide. After fermentation, the contents of isopentanol, propyl acetate, ethyl acetate, butyl acetate, and 2,4-heptadienal-*M* in SY were significantly higher than those in YL. This suggests that these substances play a significant role in determining the flavor characteristics of fermented bangcai. This conclusion aligns with the research results of Wu et al. (2015). In their study, they found that after fermentation, the main characteristic flavor substances of sauerkraut in Northeast China were esters and aldehydes, such as ethyl acetate, propyl acetate, and ethyl butyrate. After air drying, the contents of 2-methylpropanoic acid, octanal, dimethyl trisulfide, heptanal, and 3-methylbutanal in sample GY were also significantly higher than those in YL. These compounds are characteristic flavor substances of bangcai after fermentation and drying. When sample SY after fermentation was used as a reference, 50 different volatile metabolites were detected in the reference group SY and the air-dried sample GY. Among these, 28 metabolites were upregulated, and 22 metabolites were downregulated. The data showed that the volatile substances of fermented bangcai underwent complex changes during the drying process. Aldehydes, such as 2-methylbutanal, 3-methylbutanal, nonanal-*M*, octanal, and heptanal were significantly upregulated in sample GY, whereas 2,4-heptadienal-*D*, ethyl acetate, ethanol, isopentanol, propyl acetate, and butyl acetate were significantly downregulated. Therefore, aldehydes were the main flavor in sample GY, giving it a stronger flower and fruit aroma. However, alcohols, heterocycles, esters, and other substances are more susceptible to the drying process. They are reduced to varying degrees during processing (Li et al., 2023). Alcohols are converted to aldehydes under high-temperature catalysis.

### 3.2 Microbial community structure and function during the fermentation of bangcai

#### 3.2.1 Classification composition and dynamic change in the bacterial community

This study analyzed the genetic information of the microbial community during the dynamic fermentation process of bangcai using metagenomic sequencing technology, and the results are shown in Supplementary Table S3. A total of 26 phyla and 506 genera were identified in 18 samples. The relative abundance of bacteria was 98.13%, indicating that these microorganisms played a crucial role in the fermentation process of bangcai.

**Figure 4:**
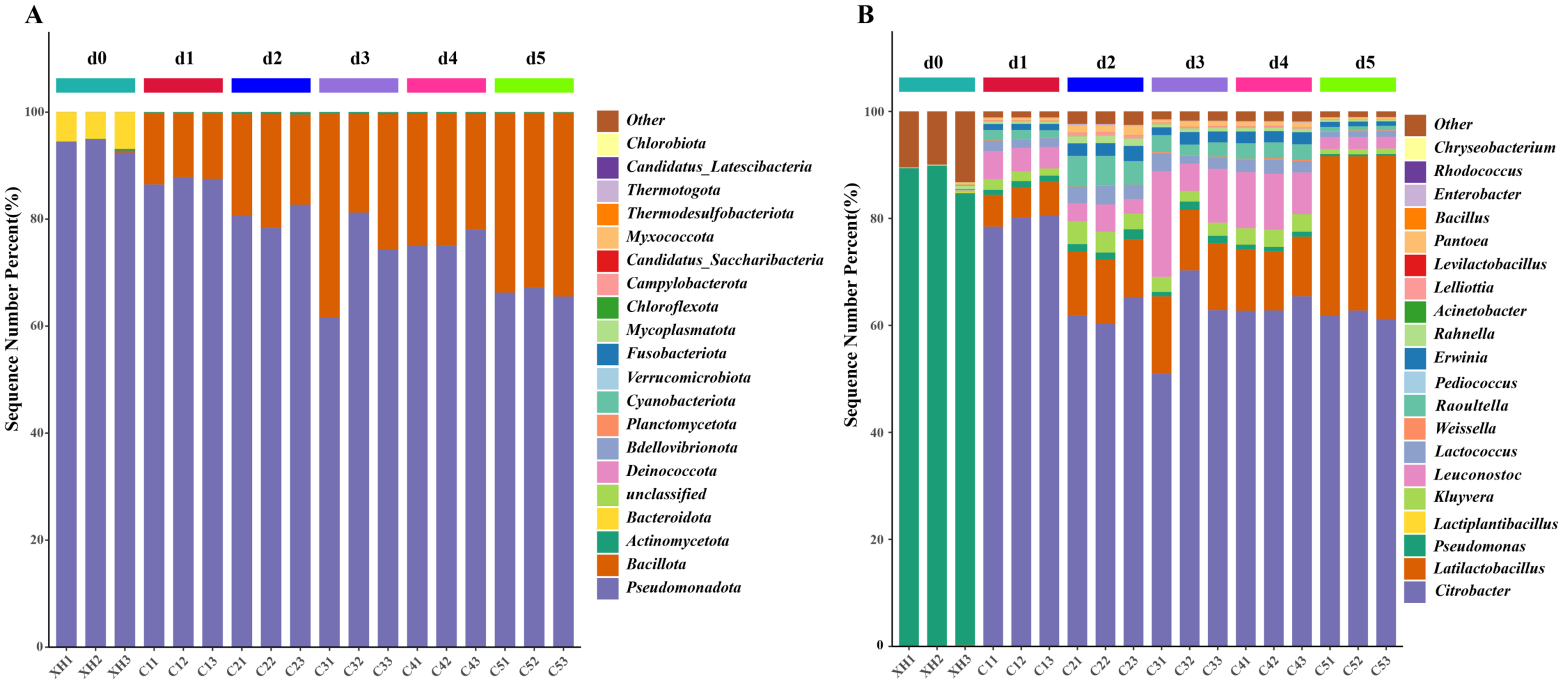
Relative abundance of bacterial community at the phylum level **(A)** genus level **(B)**. Only the top 20 species by abundance are displayed, whereas the remaining species are grouped as “others” in the figure.

As shown in Figure 4A, in the pre-fermentation stage (d0), *Pseudomonadota* was absolutely dominant (93.94%), while *Bacillota* was present in negligible amounts (0.14%), and *Bacteroidota* represented the secondary phylum (5.77%). A significant shift occurred during the mid-fermentation stage (d1–d4), where *Pseudomonadota* abundance decreased from 87.29% at d1 to 76.04% at d4. In contrast, *Bacillota* abundance increased from 12.57% at d1 to 23.72% at d4. By the late fermentation stage (d5), *Pseudomonadota* abundance further declined to 66.31%, while *Bacillota* abundance rose to 33.54%, becoming the dominant bacterial group. Throughout the entire process, other phyla, such as *Actinomycetota*, remained at low levels (0.13–0.39%), reflecting the typical microbial succession observed in fermented vegetables (Yasir et al., 2022).

At the genus level (Figure 4B), the microbial succession across the three fermentation stages became more evident. During the pre-fermentation stage (d0), *Pseudomonas* was overwhelmingly dominant (87.77%), which is consistent with the typical microbial composition on fresh vegetable surfaces. Meanwhile, *Citrobacter* content was relatively low (0.18%), and other genera such as *Rahnella* and *Pantoea* were also present in small proportions.

During the mid-fermentation stage (d1–d4), the microbial community underwent a significant transformation. The abundance of *Pseudomonas* dropped dramatically to less than 1%, while *Citrobacter* rapidly became the dominant genus, reaching its highest relative abundance at d1 (79.67%). Simultaneously, LAB increased significantly, with the abundance of *Latilactobacillus* rising from 6.01% at d1 to 11.19% at d4, and *Leuconostoc* exhibited a similar growth trend, increasing from 4.58% at d1 to 9.58% at d4. Some *Enterobacteriaceae* genera, such as *Kluyvera*, *Raoultella*, and *Erwinia*, maintained a relative abundance of 2–5% during this period, contributing to a complex microbial structure.

In the late fermentation stage (d5), *Citrobacter* remained the dominant genus (61.92%), while *Latilactobacillus* significantly increased to 29.72%, forming a dual-dominant microbial community. Interestingly, *Leuconostoc* declined sharply to 2.24%. The mid-fermentation surge followed by the decline of *Leuconostoc* in the later stage could be attributed to its sensitivity to low pH environments (Ijoma et al., 2021). Additionally, there were also reductions in other *Enterobacteriaceae* genera. The significant increase in *Latilactobacillus* at this stage aligns with the general trend observed in fermented vegetables, where LAB gradually dominate (Yu et al., 2022a). *Latilactobacillus* has been reported to produce various aromatic compounds and organic acids (Liu et al., 2024), which may be closely related to the formation of flavor compounds in bangcai.

#### 3.2.2 Bacterial community diversity analysis

**Figure 5:**
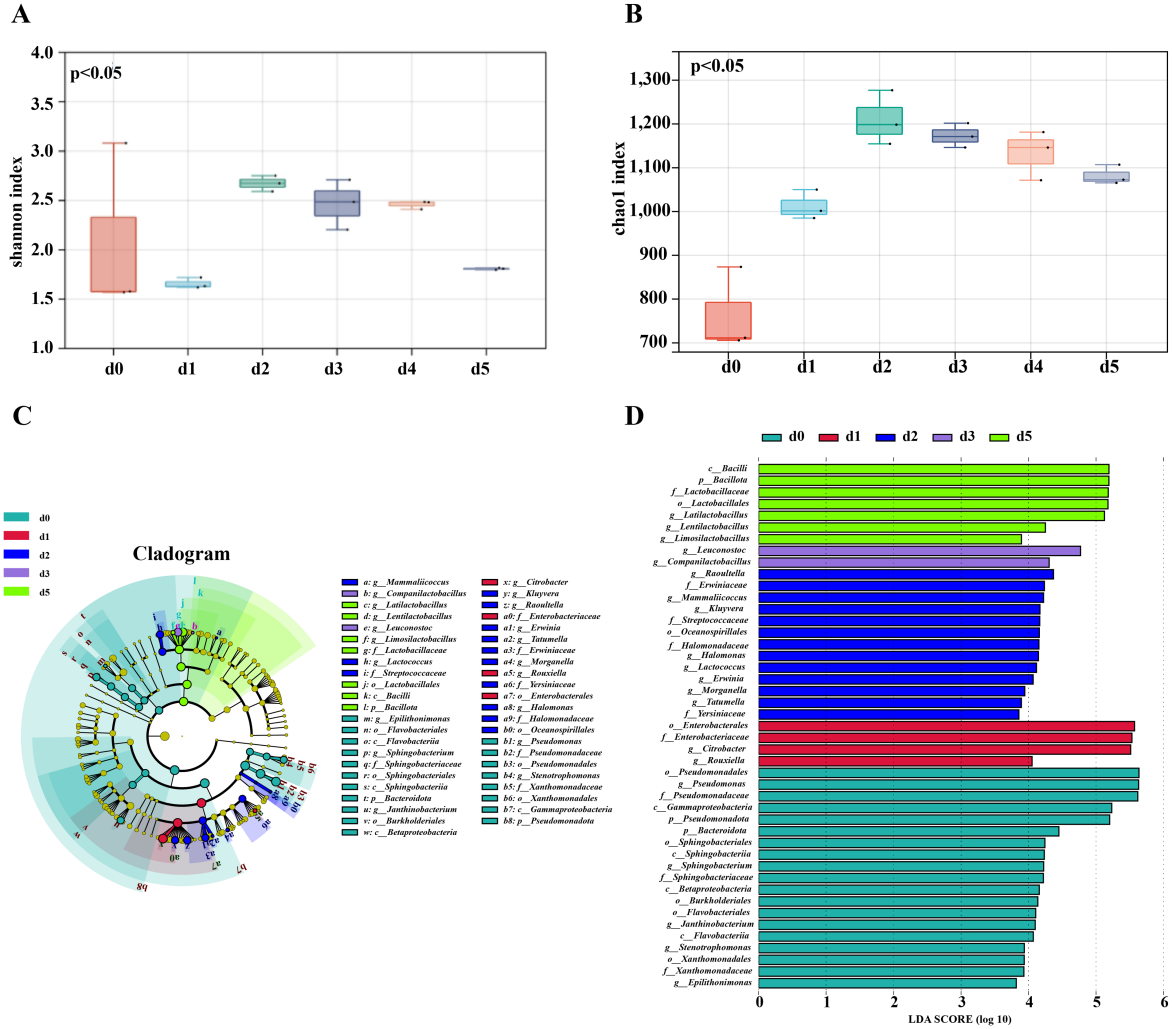
Alpha diversity index analysis of bacteria during the dynamic fermentation of bangcai: Shannon index **(A)** and Chao1 index **(B)**. In addition, LEfSe analysis was conducted to identify bacterial community differences **(C,D)** in samples before and after fermentation (LDA >3.8 and *p* < 0.05). The Tukey test was used to analyze the differences and significance of OTU diversity between groups (*p* < 0.05).

The Shannon and Chao1 indices are commonly used to evaluate the diversity and abundance of microbial communities, respectively. The greater the Shannon index, the higher the microbial diversity. In addition, the Chao1 index represents the abundance of microbial flora, and the higher the value, the higher the abundance of microbial flora. Based on the Shannon index (*p* < 0.05) and Chao1 value (*p* < 0.0001), the difference between microbial composition and relative abundance before and after fermentation was statistically significant see Figures 5A,B. The Shannon and Chao1 indices of the bacterial community before and after the fermentation of bangcai exhibited a trend of initially increasing and then slowly decreasing, with both peaking on the second day of fermentation (*p* < 0.05). The findings indicate that during the initial stage of the fermentation of bangcai, the abundance and diversity of the bacterial community gradually increased. This was attributed to the rapid propagation of bacteria using the rich nutrients in bangcai. In addition, the growth of some bacteria was inhibited by the anaerobic and high osmotic environment formed by the increase in the number of microorganisms (Dall Cortivo et al., 2020). Furthermore, the diversity and abundance of the bacterial community at the end of fermentation were higher than those at the beginning of fermentation.

LDA effective size (LEfSe) can be used to analyze differences in microorganisms between groups. Genera with LDA scores greater than 3.8 were identified as bacterial differential genera and designated as biomarkers. As can be seen from Figures 5C,D, 19 bacterial genera were identified as differential microorganisms in the fermentation process of bangcai. Of these, five different genera were enriched in the prefermentation sample d0. These genera belong to the phyla *Pseudomonadota* and *Bacteroidota*, which are often found in soil and plants and can cause a variety of human intestinal diseases (Kiely et al., 2018). Their relative content decreased after fermentation. Among them, *Pseudomonas* was the most representative genus of differential bacteria, with an LDA value of 5.63, much greater than that of *Sphingobacterium* (4.22), which was in second place and the dominant genus before the fermentation of a variety of vegetables (Yu et al., 2022b).

In the early stage of fermentation (1–2 days), 10 different genera were enriched. Seven of these genera belong to the order *Enterobacterales*. Among them, *Citrobacter* (5.51), *Morganella* (3.94), and *Tatumella* (3.89) were identified as human pathogenic bacteria (Mihu et al., 2023), this indicates that in addition to the accumulation of nitrites and biogenic amines during the early stage of fermentation (1–2 days) of bangcai, a significant number of harmful pathogenic bacteria were also thriving. However, *Lactococcus* (4.12) and *Rouxiella* (4.05) were also enriched in the early stage of fermentation, and they are commonly added as probiotics in food products (Novotny-Nuñez et al., 2023).

In the late stage of fermentation (3–5 days), five different bacterial genera, all belonging to *Lactobacillus*, were enriched, namely *Limosilactobacillus* (3.89), *Lentilactobacillus* (4.25), *Latilactobacillus* (5.12), *Leuconostoc* (4.77), and *Companilactobacillus* (4.31). These bacterial genera are often used as leavening agents in a variety of fermented foods to produce nutritious and flavorful compounds (Wang et al., 2023). Their accumulation also marks the gradual maturation of fermentation.

#### 3.2.3 KEGG functional annotations for microorganisms

**Figure 6:**
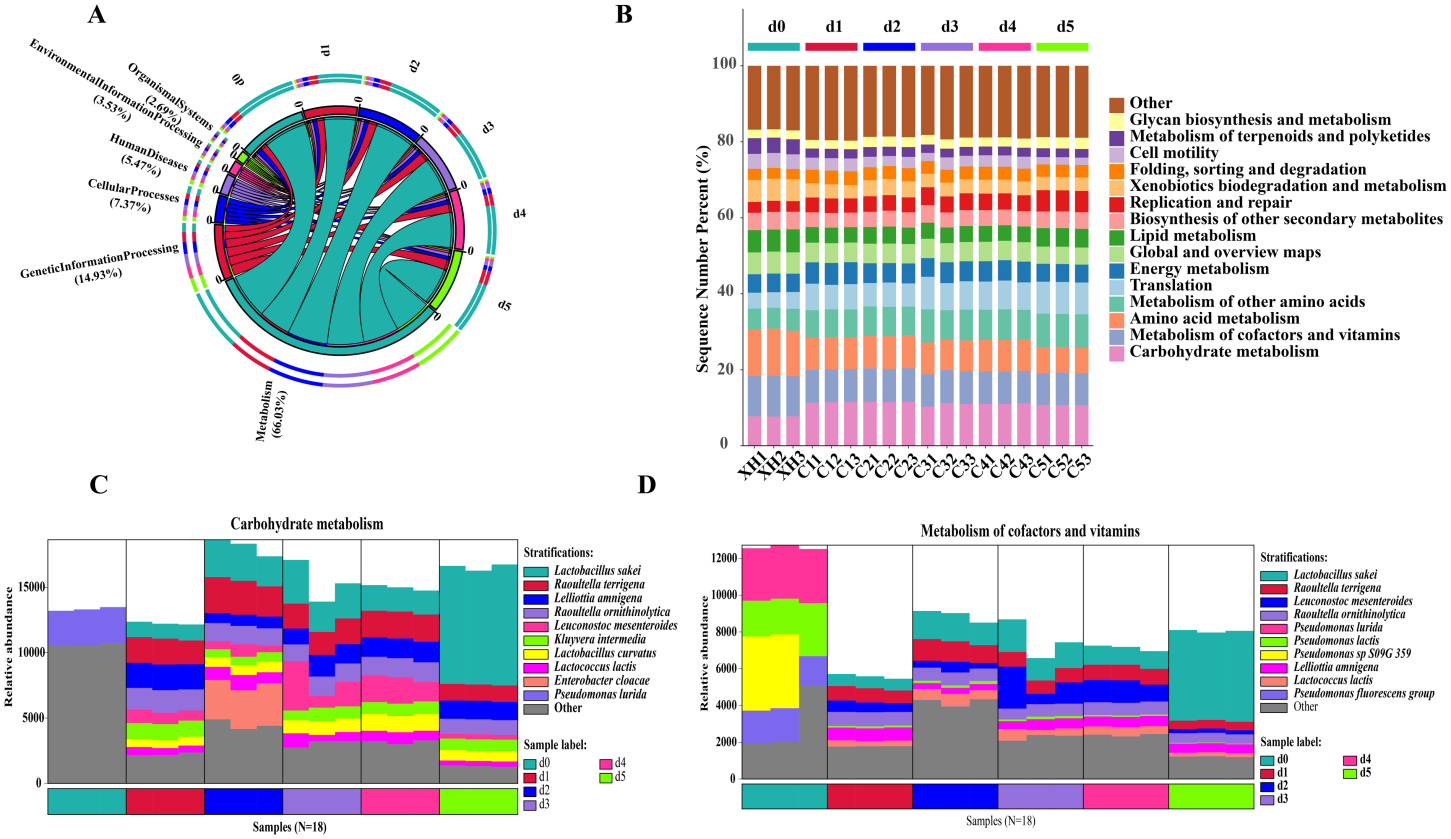
Predictive genes of the KEGG pathway at level 1 **(A)** and level 2 **(B)** in a microbial community related to “metabolism” in KEGG level 1, functional gene classification source species histogram (carbohydrate metabolism **C**, metabolism of cofactors and vitamins **D**).

To better understand the identified gene-related functional pathways in fermented bangcai, we determined the relative abundance of various functions based on the relationship between UniRef90 ID and the KEGG database. The primary KEGG metabolic pathway classification can be divided into six major categories (Figure 6A), with microbial functional genes primarily distributed across metabolism (66.03%), genetic information processing (14.93%), and cellular processes (7.37%).

KEGG metabolic pathway analysis (Figure 6B) revealed distinct functional metabolic patterns across the three fermentation stages. The pre-fermentation stage (d0) was dominated by amino acid metabolism (12.21%) and cofactor and vitamin metabolism (10.67%). At this stage, K00826 (branched-chain amino acid aminotransferase, BCAT) exhibited the highest relative abundance, primarily derived from *Sphingobacterium rhizobacter* and *Flavobacterium psychrophilum* (Supplementary Figure S2), since BCAT catalyzes the transamination of branched-chain amino acids into their corresponding α-keto acids, which act as precursors for branched-chain aldehydes, this may account for the high relative concentrations of branched-chain aldehydes detected in d0 samples. During the mid-fermentation stage (d1–d4), carbohydrate metabolism pathways were significantly enriched, peaking at d2 (11.59%), while amino acid metabolism decreased to approximately 8.30%, and other amino acid-related metabolic pathways increased to about 7.80%. In the late fermentation stage (d5), translation-related pathways reached 8.39% (compared to 4.24% at d0), and replication and repair pathways reached 5.58% (compared to 2.86% at d0); meanwhile, amino acid metabolism decreased to 6.68%, and cell motility pathways decreased to 1.94% (compared to 3.90% at d0). These sequential changes in functional pathways reflect the functional evolution of microbial communities during fermentation: transitioning from early degradation of complex organic compounds to later microbial proliferation and secondary metabolite production. The enrichment of translation and replication pathways indicates rapid proliferation of specific microbial groups (especially lactic acid bacteria) (Yao et al., 2025).

Figure 6C illustrates the microbial succession in carbohydrate metabolism pathways. The pre-fermentation stage was dominated by *Pseudomonas fluorescens*, while mid-fermentation showed a gradual increase in lactic acid bacteria (*Lactobacillus curvatus*, *Lactobacillus sakei*, and *Leuconostoc mesenteroides*). By late fermentation, *Lactobacillus sakei* became the dominant species (53–56%). Similarly, Figure 6D shows the transition in cofactor and vitamin metabolism, shifting from *Pseudomonas* dominance to *Lactobacillus sakei* predominance (55–57%) in late fermentation.

This functional transition reflects the adaptation of microbial communities to changing environmental conditions during fermentation, particularly the dominance of lactic acid bacteria in late fermentation. Xia et al. (2025) indicated that lactic acid bacteria, especially *L. sakei*, can produce various antimicrobial substances and organic acids, which not only contribute to the flavor formation of fermented foods but also inhibit pathogen growth, improving food safety. The predominant role of lactic acid bacteria in carbohydrate and vitamin metabolism suggests their significant contribution to the flavor and nutritional characteristics of bangcai.

### 3.3 Correlation analysis between microbial community structure and differential volatile compounds

**Figure 7:**
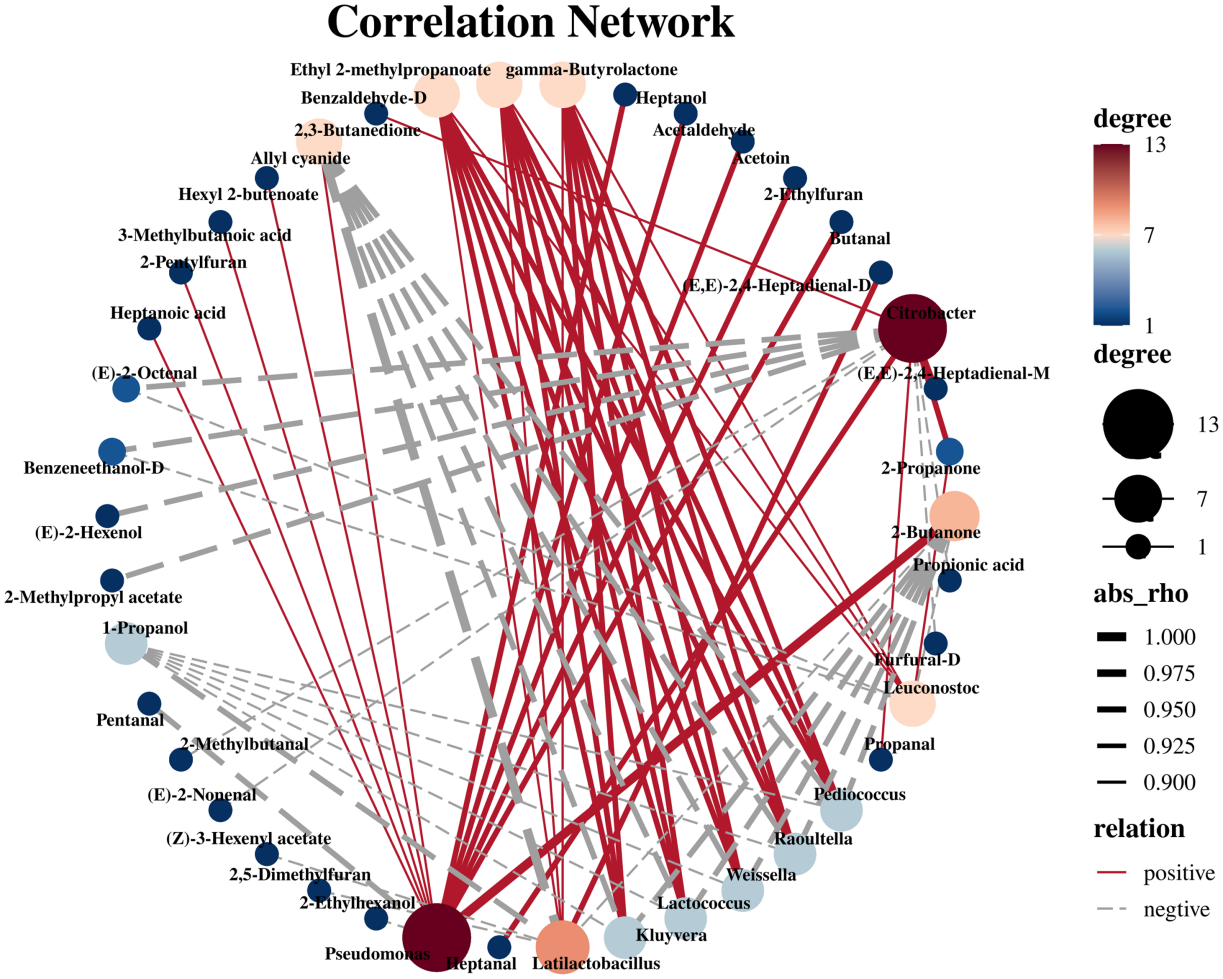
Network correlation analysis of bacteria (top 10 relative abundance) and volatile metabolite compounds (VIP >1) after fermentation (|*R*| > 0.50, *p* < 0.05).

To determine the biological factors influencing the difference in volatile metabolites before and after the fermentation of bangcai, we analyzed the correlation between the top 10 microorganisms in relative abundance and the differences in VOCs before and after the bangcai fermentation at the genus level. The results are shown in Figure 7, spearman correlation analysis revealed that 10 bacterial genera were strongly correlated with 34 VOCs (|*R*| > 0.50, *p* < 0.05). *Pseudomonas* is the genus with the largest number of positively correlated VOCs, significantly correlated with 11 VOCs and negatively correlated with 2 VOCs. It has been proven to be the core microorganism in the fermentation process of fermented mare’s milk wine (Meng et al., 2021) and fermented chili (Chen et al., 2022). In this study, *Pseudomonas* was significantly positively correlated with 2-butanone (*R* = 1, *p* < 0.001) and hexanoic acid (*R* = 0.88). At low concentrations, 2-butanone imparts a pleasant fruity and sweet aroma to the food, while hexanoic acid enhances the complexity of the flavor with a mild fatty scent. These characteristics contribute to improving the flavor of the fermented bangcai. However, at high concentrations, 2-butanone may release a pungent chemical odor (Zhang et al., 2020), and hexanoic acid may emit a strong rancid fatty odor, leading to a decline in the overall flavor (Solomando et al., 2020). Therefore, it is crucial to strictly control the levels of *Pseudomonas* and its metabolites during the fermentation process to fully harness its positive contributions to flavor while avoiding the production of undesirable odors.

Lactic acid bacteria (*Latilactobacillus* and *Leuconostoc*) are well-established as core microorganisms in fermented foods. These bacteria metabolize substrates such as sugars and organic acids, producing significant amounts of lactic acid, acetic acid, and other metabolites, which help regulate the pH of the fermentation medium, creating a favorable environment for the metabolism of other microorganisms (Suzuki et al., 2020). Lactic acid, as one of the main metabolic products of lactic acid bacteria, also serves as an important precursor for the synthesis of numerous secondary metabolites (Tang et al., 2019). Through complex metabolic pathways, these bacteria can synthesize various VOCs, such as γ-butyrolactone (*R* = 0.88), ethyl 2-methylpropanoate (*R* = 0.88), and benzaldehyde-*D* (*R* = 0.88), as well as other complex flavor compounds. The relative contents of these compounds gradually increased during fermentation, resulting in aroma, fruit aroma, and nut flavor, respectively. The overall flavor is similar to that of processed Daocai (Liu et al., 2022). In addition, 2-butanone (*R* = − 0.88) was significantly negatively correlated with both microorganisms. *Latilactobacillus* was also significantly positively correlated with (*E*,*E*)-2,4-heptadienal-*D* (*R* = 0.94). This VOC enabled the bangcai to retain some green grass fragrance even after fermentation. Similarly, microorganisms such as *Pediococcus*, *Raoultella*, *Weissella*, and *Lactococcus* showed a positive correlation with γ-butyrolactone (*R* = 0.94), ethyl 2-methylpropanoate (*R* = 0.94), and benzaldehyde-*D* (*R* = 0.94). Moreover, they were significantly negatively correlated with allyl cyanide (*R* = −0.94), 2-butanone (*R* = −0.94), and 1-propanol (*R* = −0.88). These bacterial genera have been confirmed as the primary bacterial genera in the traditional fermentation process. This study also reveals that these core species play a central role in providing flavor compounds for food. It is worth noting that *Citrobacter* was significantly negatively correlated with key VOCs such as 2-methylbutanal (*R* = −0.94), (*E*)-2-nonenal (*R* = −0.94), benzeneethanol-*D* (*R* = −0.94), 2-methylpropyl acetate (*R* = −0.94), (*E*)-2-octanal (*R* = −0.88), and (*E*)-2-hexenol (*R* = −0.88). In contrast, it was positively correlated with only 2-propanone (*R* = 0.94), (*E*,*E*)-2,4-heptadienal (*R* = 0.94), and 2,3-butanedione (*R* = 0.88). As stated in section 3.2.2, *Citrobacter* was found to be significantly enriched in the intestine of patients with gastric cancer and is one of the health hazards in fermented vegetables (Amaretti et al., 2020). At present, there are few studies on the effect of *Citrobacter* on the flavor quality of fermented vegetables. However, the negative impact of *Citrobacter* in the fermentation process cannot be ignored. For instance, in this study, *Citrobacter* showed a negative correlation with several key flavor compounds, including Benzeneethanol-*D* and (*E*)-2-Hexenol, both of which play a vital role in defining the desirable flavor profile of the fermented product. Therefore, to improve the flavor quality of fermented food and ensure food safety, it is essential to improve the processing technology to mitigate its effects, which is also the focus of our future research.

### 3.4 Association network of microbial and ester flavor compounds in traditional fermented bangcai

Based on the above discussion results, the primary flavor compounds in SY samples are contributed by various esters, including ethyl acetate, propyl acetate, and others. Correlation analysis indicates that certain esters (such as γ-butyrolactone and ethyl 2-methylpropanoate) increase with fermentation time under the regulation of LABs. Therefore, this section will focus on the relationship between ester production and LABs during bangcai fermentation.

**Figure 8:**
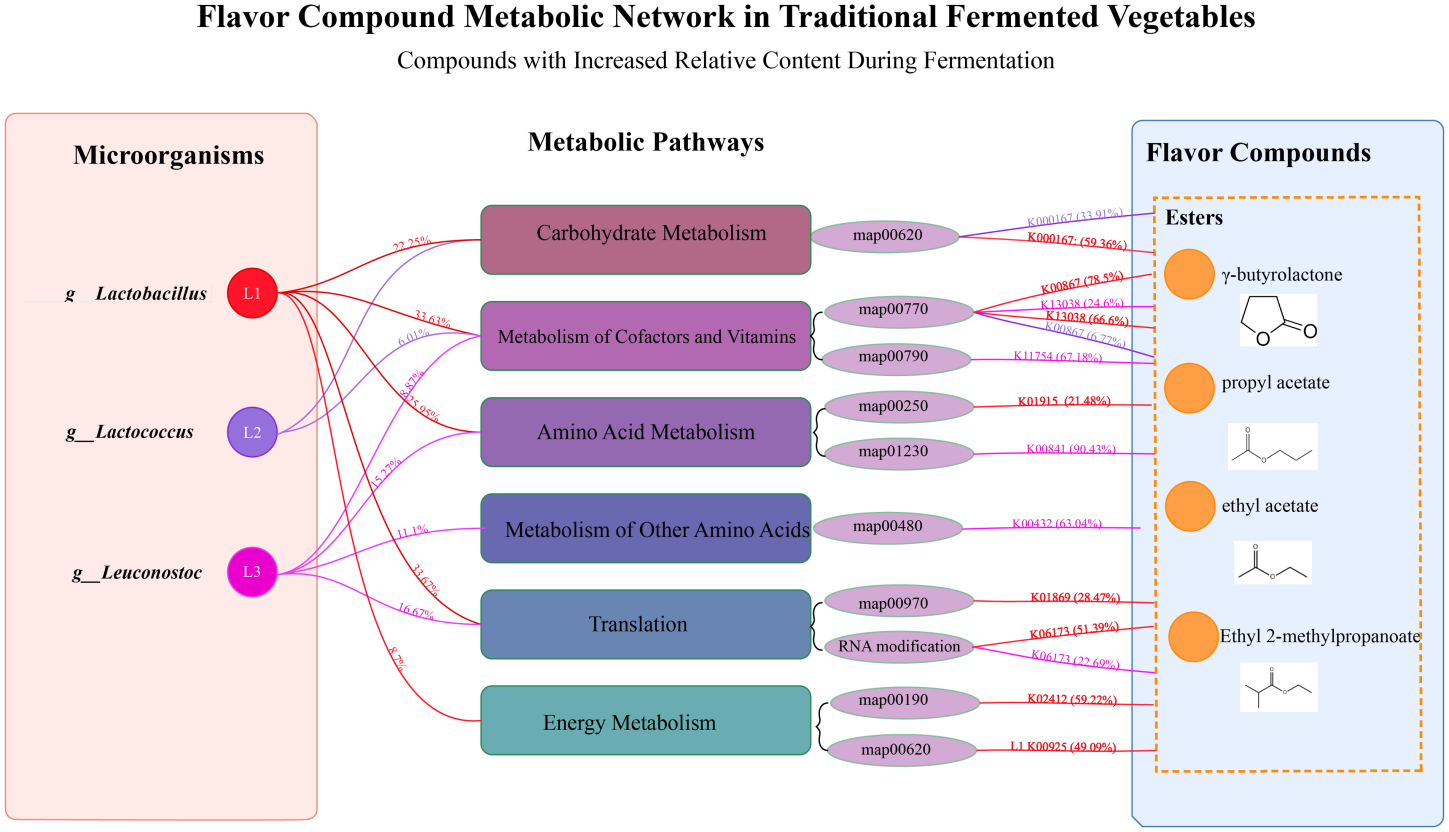
The flavor compound metabolic network diagram of traditional fermented bangcai. This diagram shows the relationships between LABs (on the left), metabolic pathways (in the middle), and the ester flavor compounds produced (on the right) during fermentation. The percentages on the left indicate the relative contribution of each LABs to the KEGG secondary metabolic pathways, while the percentages on the right indicate the contribution of microorganisms to specific functional gene clusters. Lines of the same color represent contributions from the same microbial genus.

To visually demonstrate the complex interactions among LABs, metabolic pathways, and flavor compounds, a metabolic network diagram has been constructed. As shown in Figure 8, the formation of esters during bangcai fermentation results from the synergistic action of various LABs. The three main LABs shown on the left—*Lactobacillus* (L1), *Lactococcus* (L2) and *Leuconostoc* (L3)—collectively participate in ester generation through different metabolic pathways. *Lactobacillus* contributes 22.25% to carbohydrate metabolism, 33.63% to cofactor and vitamin metabolism, and 25.95% to amino acid metabolism. *Lactococcus* contributes 6.01% to cofactor and vitamin metabolism, while *Leuconostoc* contributes 11.1% to metabolism of other amino acids.

The colored lines on the right side of Figure 8 represent selected examples of functional gene connections, highlighting key enzymes involved in flavor compound formation rather than representing the complete metabolic contribution. For instance, red lines illustrate specific functional genes where *Lactobacillus* shows notable involvement: this genus accounts for 59.36% of the observed expression of K00167, 21.48% of K01915, and 28.47% of K01869, which participate in carbohydrate metabolism, amino acid metabolism, and translation processes, respectively. This finding aligns with Liu et al. (2024), who confirmed that lactic acid bacteria play a key role in the formation of ester flavor compounds in fermented vegetables through carbohydrate and amino acid metabolism pathways. Purple lines indicate that *Leuconostoc* accounts for 90.43% of the total expression of K00841, demonstrating its significant influence in amino acid metabolism.

By analyzing the contributions of LABs in different metabolic pathways and their corresponding enzymes, we can observe the complex network of ester formation during bangcai fermentation. This collaborative action of various LABs across different metabolic pathways promotes the generation and accumulation of characteristic flavor esters, which gradually increase as fermentation progresses, forming the unique flavor characteristics of bangcai.

## 4 Conclusion

In this study, aldehydes are identified as the most abundant VOCs during the fermentation process of bangcai. The main VOCs in the raw material of bangcai are esters such as allyl isothiocyanate, 1-butene isothiocyanate, and sec-butyl isothiocyanate. After fermentation, the content of 2-methyl-3-furanthiol, ethyl acetate, propyl acetate, and other substances increased, whereas the content of isothiocyanates decreased. The content of γ-butyrolactone, nonanal, phenylacetaldehyde, octanal, and other VOCs increased after drying. Microorganisms mainly include *Proteus* and *Pseudomonas*. At the genus level, *Citrobacter*, *Latilactobacillus*, and *Leuconostoc* were the dominant bacteria in the fermentation process. LEfSe analysis showed that *Sphingobacterium*, *Lactococcus*, *Rouxiella*, *Lentilactobacillus*, *Latilactobacillus*, and *Leuconostoc* were the major differential bacterial genera before and after bangcai fermentation. Notably, *Latilactobacillus* and *Leuconostoc* were strongly associated with key flavor compounds such as γ-butyrolactone, ethyl 2-methylpropanoate, and benzaldehyde-*D*. These microorganisms could be prioritized in starter culture development to optimize flavor development in fermentation. However, while *Pseudomonas* contributed precursors for VOCs synthesis, it also produced undesirable off-flavor compounds like 2-butanone and heptanoic acid, warranting careful management of its role in the fermentation process. This study highlights the pivotal role of microorganisms in shaping volatile profiles, offering valuable insights for starter development and the targeted screening of functional microbes.

## Data Availability

The original contributions presented in the study are included in the article/Supplementary material, further inquiries can be directed to the corresponding author.

## Footnotes

^1^ http://bowtie-bio.sourceforge.net/bowtie2/index.shtml

^2^ https://www.metaboanalyst.ca/
