# Supplementary material for: Changes in microbial community succession and volatile compounds during the natural fermentation of bangcai
Source: Front Microbiol. 2025 Apr 2;16:1581378. doi: 10.3389/fmicb.2025.1581378 (PMC11999953; doi:10.3389/fmicb.2025.1581378)
Supplement: Supplementary file 1 [file Table_1.docx]

**Appendix A. Supplementary tables**

**Table S1** VOCs detected by GC-IMS in bangcai.

| No. | VOCs | CAS# | Molecular Formula | MW | RI | Rt [sec] | Dt [a.u.] |
| --- | --- | --- | --- | --- | --- | --- | --- |
| 1 | Allyl Isothiocyanate | C57067 | C4H5NS | 99.2 | 887.3 | 254.142 | 1.08982 |
| 2 | Allyl Isothiocyanate | C57067 | C4H5NS | 99.2 | 885 | 252.944 | 1.37817 |
| 3 | Hexyl 2-butenoate | C19089920 | C10H18O2 | 170.3 | 1334.4 | 830.11 | 1.44766 |
| 4 | Decanal | C112312 | C10H20O | 156.3 | 1273.5 | 742.686 | 1.53845 |
| 5 | Diethyl succinate | C123251 | C8H14O4 | 174.2 | 1191.5 | 624.854 | 1.30358 |
| 6 | (E)-2-Nonenal | C18829566 | C9H16O | 140.2 | 1164.6 | 586.277 | 1.40106 |
| 7 | Nonanal | C124196 | C9H18O | 142.2 | 1108.4 | 505.576 | 1.47465 |
| 8 | Nonanal | C124196 | C9H18O | 142.2 | 1108.6 | 505.938 | 1.95195 |
| 9 | Linalool | C78706 | C10H18O | 154.3 | 1097 | 489.239 | 1.24589 |
| 10 | 4-pentenyl isothiocyanate | R283637 | C6H9NS | 127.2 | 1079.3 | 463.768 | 1.17398 |
| 11 | Benzeneacetaldehyde | C122781 | C8H8O | 120.2 | 1038.9 | 405.757 | 1.25359 |
| 12 | Benzeneacetaldehyde | C122781 | C8H8O | 120.2 | 1039.7 | 406.929 | 1.53783 |
| 13 | (Z)-3-Hexenyl acetate | C3681718 | C8H14O2 | 142.2 | 1009.9 | 364.153 | 1.81426 |
| 14 | 2,4,5-Trimethylthiazole | C13623115 | C6H9NS | 127.2 | 983.4 | 333.858 | 1.56086 |
| 15 | 1-Butene 4-isothiocyanate | C3386978 | C5H7NS | 113.2 | 984.8 | 335.029 | 1.13312 |
| 16 | sec-Butyl Isothiocyanate | C4426793 | C5H9NS | 115.2 | 929.4 | 288.19 | 1.14337 |
| 17 | (E)-2-Hexenol | C928950 | C6H12O | 100.2 | 851.1 | 235.313 | 1.51049 |
| 18 | Furfural | C98011 | C5H4O2 | 96.1 | 825.2 | 221.866 | 1.09004 |
| 19 | Furfural | C98011 | C5H4O2 | 96.1 | 825.9 | 222.24 | 1.32534 |
| 20 | Hexanal | C66251 | C6H12O | 100.2 | 789.3 | 203.191 | 1.5632 |
| 21 | (E)-2-Pentenal | C1576870 | C5H8O | 84.1 | 743.9 | 184.156 | 1.10519 |
| 22 | (E)-2-Pentenal | C1576870 | C5H8O | 84.1 | 744.6 | 184.429 | 1.36059 |
| 23 | 2-Pentenal | C764396 | C5H8O | 84.1 | 730.5 | 178.694 | 1.36384 |
| 24 | 2,5-Dimethylfuran | C625865 | C6H8O | 96.1 | 692.8 | 163.348 | 1.35955 |
| 25 | Acetoin | C513860 | C4H8O2 | 88.1 | 687.2 | 161.264 | 1.33615 |
| 26 | 2-Ethylfuran | C3208160 | C6H8O | 96.1 | 675.4 | 158.138 | 1.30901 |
| 27 | Allyl cyanide | C109751 | C4H5N | 67.1 | 643.2 | 149.629 | 1.24536 |
| 28 | Butanal | C123728 | C4H8O | 72.1 | 601.1 | 138.515 | 1.28654 |
| 29 | 2-Butanone | C78933 | C4H8O | 72.1 | 568.2 | 129.832 | 1.2463 |
| 30 | Methyl acetate | C79209 | C3H6O2 | 74.1 | 512.9 | 115.245 | 1.19388 |
| 31 | Propanal | C123386 | C3H6O | 58.1 | 495.9 | 110.73 | 1.15644 |
| 32 | Acetaldehyde | C75070 | C2H4O | 44.1 | 421.6 | 91.107 | 0.98703 |
| 33 | 1-Pentanol | C71410 | C5H12O | 88.1 | 759.4 | 190.438 | 1.2491 |
| 34 | gamma-Butyrolactone | C96480 | C4H6O2 | 86.1 | 916.8 | 277.527 | 1.30081 |
| 35 | Octanal | C124130 | C8H16O | 128.2 | 1004.6 | 356.597 | 1.40453 |
| 36 | (E,E)-2,4-Hexadienal | C142836 | C6H8O | 96.1 | 908 | 270.01 | 1.45062 |
| 37 | Heptanol | C53535334 | C7H16O | 116.2 | 959.5 | 313.62 | 1.38444 |
| 38 | Heptanal | C111717 | C7H14O | 114.2 | 900.7 | 263.883 | 1.70185 |
| 39 | 3-Methylbutanoic acid | C503742 | C5H10O2 | 102.1 | 899 | 262.441 | 1.2048 |
| 40 | 2-Methyl-3-furanthiol | C28588741 | C5H6OS | 114.2 | 865.1 | 242.619 | 1.14267 |
| 41 | (E)-2-Hexenal | C6728263 | C6H10O | 98.1 | 836 | 227.482 | 1.17373 |
| 42 | Butyl acetate | C123864 | C6H12O2 | 116.2 | 802.7 | 210.182 | 1.62486 |
| 43 | Propyl acetate | C109604 | C5H10O2 | 102.1 | 703.4 | 167.653 | 1.48034 |
| 44 | 2-Methylbutanal | C96173 | C5H10O | 86.1 | 659.7 | 154.001 | 1.40559 |
| 45 | Ethyl Acetate | C141786 | C4H8O2 | 88.1 | 596.5 | 137.296 | 1.34066 |
| 46 | 2-Propanone | C67641 | C3H6O | 58.1 | 463.3 | 102.14 | 1.119 |
| 47 | Ethanol | C64175 | C2H6O | 46.1 | 442.5 | 96.655 | 1.04735 |
| 48 | 2,3-Butanedione | C431038 | C4H6O2 | 86.1 | 547.2 | 124.276 | 1.15823 |
| 49 | 3-Methylbutanal | C590863 | C5H10O | 86.1 | 631.2 | 146.468 | 1.41457 |
| 50 | 2-Methylpropanoic acid | C79312 | C4H8O2 | 88.1 | 770.8 | 195.113 | 1.38153 |
| 51 | Methyl butanoate | C623427 | C5H10O2 | 102.1 | 724.2 | 176.117 | 1.41758 |
| 52 | gamma-Octalactone | C104507 | C8H14O2 | 142.2 | 1283.2 | 756.586 | 1.33246 |
| 53 | (E,E)-2,4-Heptadienal | C4313035 | C7H10O | 110.2 | 1010.2 | 364.619 | 1.19471 |
| 54 | 2,4-Heptadienal | C5910850 | C7H10O | 110.2 | 1000.8 | 351.101 | 1.20339 |
| 55 | Trimethylpyrazine | C14667551 | C7H10N2 | 122.2 | 1000.5 | 350.665 | 1.1761 |
| 56 | Benzaldehyde | C100527 | C7H6O | 106.1 | 957.9 | 312.29 | 1.14633 |
| 57 | 2-Methylbutanoic acid | C116530 | C5H10O2 | 102.1 | 869.1 | 244.697 | 1.47503 |
| 58 | Propionic acid | C79094 | C3H6O2 | 74.1 | 706.7 | 168.978 | 1.1019 |
| 59 | Hexanoic acid | C142621 | C6H12O2 | 116.2 | 1013.4 | 369.225 | 1.64815 |
| 60 | (E)-2-Heptenal | C18829555 | C7H12O | 112.2 | 954.7 | 309.558 | 1.66967 |
| 61 | (E)-2-Octenal | C2548870 | C8H14O | 126.2 | 1054.8 | 428.592 | 1.33347 |
| 62 | alpha-Phellandrene | C99832 | C10H16 | 136.2 | 1003.6 | 355.158 | 1.22487 |
| 63 | (E,E)-2,4-Heptadienal | C4313035 | C7H10O | 110.2 | 1011.2 | 366 | 1.6227 |
| 64 | 2,4-Heptadienal | C5910850 | C7H10O | 110.2 | 1000.5 | 350.723 | 1.63033 |
| 65 | 2-Ethylhexanol | C104767 | C8H18O | 130.2 | 1030.7 | 394.092 | 1.399 |
| 66 | Benzaldehyde | C100527 | C7H6O | 106.1 | 959.2 | 313.37 | 1.46708 |
| 67 | Ethanol | C64175 | C2H6O | 46.1 | 442.5 | 96.645 | 1.1368 |
| 68 | Tetrahydrofuran | C109999 | C4H8O | 72.1 | 640.9 | 149.023 | 1.22725 |
| 69 | Methyl isobutyrate | C547637 | C5H10O2 | 102.1 | 653.4 | 152.344 | 1.44375 |
| 70 | 2-Methylpropyl acetate | C110190 | C6H12O2 | 116.2 | 760.8 | 191.027 | 1.61692 |
| 71 | 1-Pentanol | C71410 | C5H12O | 88.1 | 760.8 | 191.027 | 1.51815 |
| 72 | Ethyl 2-methylpropanoate | C97621 | C6H12O2 | 116.2 | 749.8 | 186.564 | 1.57552 |
| 73 | 2,3-Butanediol | C513859 | C4H10O2 | 90.1 | 776 | 197.196 | 1.36153 |
| 74 | 2-Methyl propanal | C78842 | C4H8O | 72.1 | 538.9 | 122.095 | 1.28348 |
| 75 | 1-Hydroxy-2-propanone | C116096 | C3H6O2 | 74.1 | 693.7 | 163.71 | 1.04544 |
| 76 | Dimethyl disulfide | C624920 | C2H6S2 | 94.2 | 735.5 | 180.717 | 1.14267 |
| 77 | 2-Methyl-2-propenal | C78853 | C4H6O | 70.1 | 547.1 | 124.266 | 1.22201 |
| 78 | (Z)-2-Pentenol | C1576950 | C5H10O | 86.1 | 777.3 | 197.725 | 1.44552 |
| 79 | 1-Propanol | C71238 | C3H8O | 60.1 | 514.2 | 115.582 | 1.24213 |
| 80 | Ethyl 2-phenylacetate | C101973 | C10H12O2 | 164.2 | 1238.9 | 692.9 | 1.29671 |
| 81 | Methyl salicylate | C119368 | C8H8O3 | 152.1 | 1249 | 707.46 | 1.21004 |
| 82 | Isoamyl acetate | C123922 | C7H14O2 | 130.2 | 872.9 | 246.646 | 1.74852 |
| 83 | 1-Hexanol | C111273 | C6H14O | 102.2 | 872.9 | 246.646 | 1.64006 |
| 84 | 1-Octen-3-one | C4312996 | C8H14O | 126.2 | 979.4 | 330.435 | 1.69102 |
| 85 | 2-Pentylfuran | C3777693 | C9H14O | 138.2 | 993.2 | 342.138 | 1.25459 |
| 86 | Dimethyl trisulphide | C3658808 | C2H6S3 | 126.3 | 966.6 | 319.669 | 1.30556 |
| 87 | 2,5-Dimethylpyrazine | C123320 | C6H8N2 | 108.1 | 908 | 270.051 | 1.11086 |
| 88 | Benzeneethanol | C60128 | C8H10O | 122.2 | 1061.7 | 438.566 | 1.2938 |
| 89 | Benzeneethanol | C60128 | C8H10O | 122.2 | 1060.7 | 437.116 | 1.5189 |
| 90 | Heptanoic acid | C111148 | C7H14O2 | 130.2 | 1080.9 | 466.093 | 1.36907 |
| 91 | Pentanal | C110623 | C5H10O | 86.1 | 690.4 | 162.359 | 1.42234 |
| 92 | Isopentanol | C123513 | C5H12O | 88.1 | 725.9 | 176.819 | 1.50471 |

Note: MW: Molecular Weight, RI: Retention Index, Rt: Retention time; Dt: Drift time.

**Table S2 Relative concentration (peak area) of volatile organic compounds (VOCs) in Bangcai under different processing methods**.

| **Sample** | **RI** | **Rt [sec]** | **Dt [RIPrel]** | **YL-1** | **YL-2** | **YL-3** | **SY-1** | **SY-2** | **SY-3** |
| --- | --- | --- | --- | --- | --- | --- | --- | --- | --- |
| AllylIsothiocyanate-M | 887.3 | 254.14 | 1.08982 | 846.8005 | 839.66846 | 914.3436 | 721.27423 | 745.9459 | 756.75665 |
| AllylIsothiocyanate-D | 885 | 252.94 | 1.37817 | 4659.6323 | 5055.693 | 5063.13 | 3375.55 | 3678.755 | 3942.5193 |
| Hexyl2-butenoate | 1334.4 | 830.11 | 1.44766 | 647.06244 | 735.40497 | 677.27277 | 188.8543 | 155.73338 | 179.53854 |
| Decanal | 1273.5 | 742.69 | 1.53845 | 287.99805 | 265.47202 | 367.20258 | 272.81036 | 249.33846 | 250.58585 |
| Diethylsuccinate | 1191.5 | 624.85 | 1.30358 | 353.0655 | 405.60825 | 391.26166 | 223.47018 | 205.8861 | 295.67917 |
| (E)-2-Nonenal | 1164.6 | 586.28 | 1.40106 | 495.41086 | 508.76395 | 346.11755 | 107.96118 | 124.09791 | 125.4056 |
| Nonanal-M | 1108.4 | 505.58 | 1.47465 | 1456.5903 | 1387.1396 | 1472.5525 | 961.2652 | 871.2182 | 960.1797 |
| Nonanal-D | 1108.6 | 505.94 | 1.95195 | 198.36366 | 185.3978 | 195.24678 | 188.1211 | 176.87553 | 194.5231 |
| Linalool | 1097 | 489.24 | 1.24589 | 617.91223 | 674.30505 | 670.674 | 118.10218 | 134.28651 | 197.64635 |
| 4-Pentenylisothiocyanate | 1079.3 | 463.77 | 1.17398 | 2014.4913 | 2269.5461 | 2175.8206 | 358.45816 | 455.11664 | 598.21423 |
| Benzeneacetaldehyde-M | 1038.9 | 405.76 | 1.25359 | 1300.9744 | 1490.7555 | 1516.811 | 221.01031 | 272.67703 | 274.80048 |
| Benzeneacetaldehyde-D | 1039.7 | 406.93 | 1.53783 | 741.3531 | 1063.4497 | 892.8746 | 115.9121 | 124.523224 | 160.98004 |
| (Z)-3-Hexenylacetate | 1009.9 | 364.15 | 1.81426 | 15406.918 | 15849.713 | 12274.034 | 436.29788 | 503.54904 | 466.19397 |
| 2,4,5-Trimethylthiazole | 983.4 | 333.86 | 1.56086 | 545.4493 | 741.6896 | 608.2505 | 123.32027 | 143.97046 | 148.57278 |
| 1-Butene4-isothiocyanate | 984.8 | 335.03 | 1.13312 | 4778.217 | 4973.2354 | 5060.8955 | 2821.407 | 3555.8186 | 3665.3223 |
| sec-ButylIsothiocyanate | 929.4 | 288.19 | 1.14337 | 3592.9927 | 4140.994 | 4219.0557 | 2628.4265 | 2769.4292 | 3287.4646 |
| (E)-2-Hexenol | 851.1 | 235.31 | 1.51049 | 14229.321 | 13891.043 | 13159.107 | 9676.415 | 10017.931 | 11083.944 |
| Furfural-M | 825.2 | 221.87 | 1.09004 | 600.7757 | 770.9318 | 752.0178 | 81.66123 | 82.448395 | 136.65434 |
| Furfural-D | 825.9 | 222.24 | 1.32534 | 626.5583 | 677.2601 | 543.48456 | 196.38942 | 208.14601 | 243.80296 |
| Hexanal | 789.3 | 203.19 | 1.5632 | 536.46045 | 651.1252 | 822.08435 | 696.92316 | 727.2541 | 732.469 |
| (E)-2-Pentenal-M | 743.9 | 184.16 | 1.10519 | 417.7076 | 393.19147 | 442.36026 | 108.07545 | 117.82604 | 118.3561 |
| (E)-2-Pentenal-D | 744.6 | 184.43 | 1.36059 | 2241.3164 | 2357.0444 | 2035.6621 | 2189.9924 | 2256.9421 | 2448.561 |
| 2-Pentenal | 730.5 | 178.69 | 1.36384 | 240.57498 | 180.12256 | 189.0987 | 163.47482 | 137.24788 | 100.07691 |
| 2,5-Dimethylfuran | 692.8 | 163.35 | 1.35955 | 1504.9878 | 1750.6952 | 1350.9398 | 691.19403 | 703.1474 | 701.14777 |
| Acetoin | 687.2 | 161.26 | 1.33615 | 938.87885 | 1109.2794 | 941.95447 | 432.27002 | 419.2375 | 340.76614 |
| 2-Ethylfuran | 643.2 | 149.63 | 1.24536 | 2553.856 | 2746.935 | 2583.787 | 1957.0161 | 1821.114 | 1819.1302 |
| Allylcyanide | 601.1 | 138.52 | 1.28654 | 1832.6643 | 1825.2594 | 1697.4447 | 374.17908 | 385.64047 | 361.99084 |
| Butanal | 568.2 | 129.83 | 1.2463 | 1420.1526 | 1493.9613 | 1464.792 | 607.32367 | 590.07605 | 421.6307 |
| 2-Butanone | 512.9 | 115.25 | 1.19388 | 433.69516 | 548.88043 | 425.83313 | 212.52298 | 208.79985 | 145.21468 |
| Methylacetate | 495.9 | 110.73 | 1.15644 | 5178.585 | 6471.567 | 5807.098 | 5906.9976 | 5445.1074 | 5882.691 |
| Propanal | 421.6 | 91.11 | 0.98703 | 1020.5337 | 1199.9517 | 1115.6687 | 1699.0792 | 1500.7886 | 1495.8118 |
| Acetaldehyde | 759.4 | 190.44 | 1.2491 | 4057.5425 | 4512.021 | 4164.891 | 2627.2776 | 2169.0757 | 2158.9824 |
| gamma-Butyrolactone | 916.8 | 277.53 | 1.30081 | 861.69617 | 887.98975 | 870.57385 | 1952.0869 | 2120.8242 | 2332.998 |
| Octanal | 1004.6 | 356.60 | 1.40453 | 103.77464 | 110.97333 | 128.3384 | 130.65544 | 111.91601 | 122.40615 |
| (E,E)-2,4-Hexadienal | 908 | 270.01 | 1.45062 | 132.36623 | 143.60861 | 126.43398 | 165.13165 | 144.53543 | 158.3456 |
| Heptanol | 959.5 | 313.62 | 1.38444 | 187.18477 | 250.55093 | 217.03961 | 142.8405 | 104.39675 | 99.362755 |
| Heptanal | 900.7 | 263.88 | 1.70185 | 37.513763 | 40.37673 | 46.347065 | 129.82384 | 115.41378 | 76.1067 |
| 3-Methylbutanoicacid | 899 | 262.44 | 1.2048 | 48.72441 | 56.07227 | 51.930172 | 36.479034 | 33.0987 | 34.057255 |
| 2-Methyl-3-furanthiol | 865.1 | 242.62 | 1.14267 | 65.27376 | 79.91552 | 73.31673 | 416.12378 | 418.99945 | 169.8927 |
| (E)-2-Hexenal | 836 | 227.48 | 1.17373 | 425.93152 | 450.6127 | 465.19733 | 221.34041 | 238.7277 | 248.94806 |
| Butylacetate | 802.7 | 210.182 | 1.62486 | 68.44143 | 70.39663 | 63.296345 | 5736.5273 | 5727.5415 | 2604.1167 |
| Propylacetate | 703.4 | 167.653 | 1.48034 | 226.24428 | 233.58261 | 202.17885 | 7078.9575 | 6805.106 | 3923.7512 |
| 2-Methylbutanal | 659.7 | 154.001 | 1.40559 | 442.76968 | 404.98932 | 415.99365 | 186.07068 | 197.39241 | 197.53842 |
| EthylAcetate | 596.5 | 137.296 | 1.34066 | 393.90564 | 347.89502 | 299.7451 | 10053.997 | 9925.303 | 10517.671 |
| 2-Propanone | 463.3 | 102.14 | 1.119 | 553.3399 | 674.3368 | 588.3906 | 2780.7446 | 2805.2703 | 2708.1199 |
| Ethanol-M | 442.5 | 96.655 | 1.04735 | 685.62683 | 574.3075 | 662.2565 | 2326.539 | 2278.97 | 2172.2212 |
| 2,3-Butanedione | 547.2 | 124.276 | 1.15823 | 205.22908 | 274.71475 | 233.6588 | 1987.0519 | 1765.2767 | 1752.9868 |
| 3-Methylbutanal | 631.2 | 146.468 | 1.41457 | 55.20576 | 59.93823 | 63.28682 | 53.520355 | 78.56021 | 91.30391 |
| 2-Methylpropanoicacid | 770.8 | 195.113 | 1.38153 | 48.708538 | 53.748886 | 38.08826 | 107.945305 | 110.84002 | 107.2502 |
| Methylbutanoate | 724.2 | 176.117 | 1.41758 | 178.56729 | 196.60843 | 148.23 | 542.0055 | 541.377 | 504.41238 |
| gamma-Octalactone | 1283.2 | 756.586 | 1.33246 | 344.57498 | 394.14368 | 313.67905 | 503.91406 | 530.70917 | 671.5278 |
| (E,E)-2,4-Heptadienal-M | 1010.2 | 364.619 | 1.19471 | 24.081305 | 26.804615 | 35.58713 | 339.13788 | 303.61423 | 289.16925 |
| 2,4-Heptadienal-M | 1000.8 | 351.101 | 1.20339 | 171.89233 | 191.71727 | 204.23561 | 278.74258 | 225.37141 | 189.55893 |
| Trimethylpyrazine | 1000.5 | 350.665 | 1.1761 | 323.57248 | 360.6768 | 339.65842 | 75.82421 | 135.7434 | 140.56155 |
| Benzaldehyde-M | 957.9 | 312.29 | 1.14633 | 127.15766 | 173.20955 | 175.90112 | 203.12152 | 232.40823 | 193.82164 |
| 2-Methylbutanoicacid | 869.1 | 244.697 | 1.47503 | 972.5488 | 1226.826 | 1228.2417 | 1306.11 | 1330.0834 | 1407.1868 |
| Propionicacid | 706.7 | 168.978 | 1.1019 | 210.57095 | 140.38698 | 163.90967 | 41.75743 | 45.975708 | 57.621193 |
| Hexanoicacid | 1013.4 | 369.225 | 1.64815 | 80.24245 | 97.10285 | 94.17005 | 74.27211 | 60.693645 | 72.70732 |
| (E)-2-Heptenal | 954.7 | 309.558 | 1.66967 | 133.22957 | 125.13264 | 134.12146 | 2868.262 | 1539.9465 | 1205.9315 |
| (E)-2-Octenal | 1054.8 | 428.592 | 1.33347 | 546.28723 | 553.6764 | 540.2471 | 381.57455 | 356.5347 | 407.25555 |
| alpha-Phellandrene | 1003.6 | 355.158 | 1.22487 | 293.10187 | 326.22913 | 252.26173 | 217.32527 | 188.91144 | 238.5817 |
| (E,E)-2,4-Heptadienal-D | 1011.2 | 366 | 1.6227 | 35.539524 | 45.87731 | 49.448086 | 530.3092 | 487.8662 | 511.10004 |
| 2,4-Heptadienal-D | 1000.5 | 350.723 | 1.63033 | 83.060974 | 82.02307 | 71.402794 | 321.8109 | 182.37929 | 151.039 |
| 2-Ethylhexanol | 1030.7 | 394.092 | 1.399 | 77.35726 | 51.406456 | 57.243484 | 150.23915 | 227.44724 | 217.2618 |
| Benzaldehyde-D | 959.2 | 313.37 | 1.46708 | 69.720566 | 87.86645 | 73.2596 | 186.21986 | 284.95416 | 334.90692 |
| Ethanol-D | 442.5 | 96.645 | 1.1368 | 31.870354 | 22.472075 | 29.502533 | 3035.079 | 2941.7373 | 2960.461 |
| Tetrahydrofuran | 640.9 | 149.023 | 1.22725 | 1101.8553 | 1036.4451 | 997.3665 | 1029.5924 | 982.3915 | 1080.0911 |
| Methylisobutyrate | 653.4 | 152.344 | 1.44375 | 45.982056 | 50.266983 | 42.093876 | 297.7518 | 373.55063 | 496.20117 |
| 2-Methylpropylacetate | 760.8 | 191.027 | 1.61692 | 78.252335 | 71.00287 | 58.2433 | 36.513947 | 38.71037 | 41.90661 |
| 1-Pentanol | 760.8 | 191.027 | 1.51815 | 106.98358 | 102.41616 | 106.55826 | 172.46048 | 185.7501 | 150.94379 |
| Ethyl2-methylpropanoate | 749.8 | 186.564 | 1.57552 | 22.335592 | 23.456022 | 23.34493 | 32.755905 | 38.853203 | 44.302998 |
| 2,3-Butanediol | 776 | 197.196 | 1.36153 | 178.3959 | 171.62888 | 136.62894 | 337.72226 | 294.48892 | 344.0703 |
| 2-Methylpropanal | 538.9 | 122.095 | 1.28348 | 97.147285 | 91.77684 | 87.41573 | 101.81944 | 102.60026 | 89.31062 |
| 1-Hydroxy-2-propanone | 693.7 | 163.71 | 1.04544 | 114.01404 | 111.81444 | 105.7711 | 107.0915 | 104.97125 | 147.69041 |
| Dimethyldisulfide | 735.5 | 180.717 | 1.14267 | 31.172068 | 31.584692 | 30.188122 | 78.66813 | 81.71837 | 91.97046 |
| 2-Methyl-2-propenal | 547.1 | 124.266 | 1.22201 | 212.46268 | 236.83916 | 206.7907 | 598.23645 | 589.17145 | 529.95056 |
| (Z)-2-Pentenol | 777.3 | 197.725 | 1.44552 | 81.61045 | 65.04523 | 53.691753 | 181.00494 | 152.8704 | 83.94335 |
| 1-Propanol | 514.2 | 115.582 | 1.24213 | 56.20875 | 45.582127 | 45.59165 | 28.639198 | 34.860283 | 26.70622 |
| Ethyl2-phenylacetate | 1238.9 | 692.9 | 1.29671 | 166.75041 | 168.93732 | 156.7808 | 228.55814 | 255.88963 | 304.12207 |
| Methylsalicylate | 1249 | 707.46 | 1.21004 | 146.20497 | 127.79564 | 157.94885 | 108.7039 | 110.99237 | 100.810104 |
| Isoamylacetate | 872.9 | 246.646 | 1.74852 | 86.60319 | 86.85711 | 88.91387 | 88.41238 | 100.886284 | 108.10084 |
| 1-Hexanol | 872.9 | 246.646 | 1.64006 | 193.36458 | 191.19673 | 178.77678 | 292.4258 | 314.09167 | 371.38913 |
| 1-Octen-3-one | 979.4 | 330.435 | 1.69102 | 34.990417 | 32.162365 | 35.526825 | 193.63437 | 95.84911 | 68.085945 |
| 2-Pentylfuran | 993.2 | 342.138 | 1.25459 | 294.016 | 298.55167 | 295.72995 | 268.2842 | 250.22084 | 254.44229 |
| Dimethyltrisulphide | 966.6 | 319.669 | 1.30556 | 49.971798 | 54.996277 | 46.9787 | 116.33425 | 158.72966 | 188.90509 |
| 2,5-Dimethylpyrazine | 908 | 270.051 | 1.11086 | 37.018616 | 37.13923 | 42.09705 | 49.765488 | 48.44827 | 59.465298 |
| Benzeneethanol-M | 1061.7 | 438.566 | 1.2938 | 654.73724 | 777.9877 | 735.9446 | 340.94708 | 348.20288 | 363.01288 |
| Benzeneethanol-D | 1060.7 | 437.116 | 1.5189 | 513.0489 | 546.0714 | 432.52078 | 229.13898 | 227.57419 | 281.14215 |
| Heptanoicacid | 1080.9 | 466.093 | 1.36907 | 65.987915 | 86.84124 | 77.78575 | 44.188732 | 52.203136 | 37.94543 |
| Pentanal | 690.4 | 162.359 | 1.42234 | 180.15749 | 129.90955 | 179.87498 | 530.6235 | 548.97565 | 673.829 |
| Isopentanol | 725.9 | 176.819 | 1.50471 | 28.102789 | 22.024538 | 20.996155 | 125.7357 | 96.98859 | 85.77159 |

**Table S3** Overview of the quality of macro-genomic data outputs

| sample ID | InsertSize(bp) | SeqStrategy | RawReads(#) | Raw Base(GB) | %GC | Raw Q20(%) | Raw Q30(%) | Clean Reads(#) | Cleaned(%) | Clean Q20(%) | Clean Q30(%) |
| --- | --- | --- | --- | --- | --- | --- | --- | --- | --- | --- | --- |
| C11 | 350 | (150:150) | 24251886 | 7.28 | 50 | 98.13 | 94.35 | 22952507 | 94.64 | 98.82 | 95.53 |
| C12 | 350 | (150:150) | 24039626 | 7.21 | 51 | 98.21 | 94.6 | 22519355 | 93.68 | 98.89 | 95.76 |
| C13 | 350 | (150:150) | 24849849 | 7.45 | 50 | 98.15 | 94.45 | 22600726 | 90.95 | 98.86 | 95.66 |
| C21 | 350 | (150:150) | 25124796 | 7.54 | 50 | 97.99 | 94.09 | 23532450 | 93.66 | 98.81 | 95.48 |
| C22 | 350 | (150:150) | 24428268 | 7.33 | 50 | 98.12 | 94.37 | 23290926 | 95.34 | 98.82 | 95.54 |
| C23 | 350 | (150:150) | 23743146 | 7.12 | 50 | 98.18 | 94.5 | 21845888 | 92.01 | 98.86 | 95.66 |
| C31 | 350 | (150:150) | 25390270 | 7.62 | 47 | 98.01 | 94.07 | 24112899 | 94.97 | 98.77 | 95.34 |
| C32 | 350 | (150:150) | 20973817 | 6.29 | 49 | 98.13 | 94.39 | 18616635 | 88.76 | 98.83 | 95.57 |
| C33 | 350 | (150:150) | 25056623 | 7.52 | 48 | 97.99 | 94.12 | 22456277 | 89.62 | 98.79 | 95.46 |
| C41 | 350 | (150:150) | 25519434 | 7.66 | 49 | 98.09 | 94.29 | 23574776 | 92.38 | 98.83 | 95.55 |
| C42 | 350 | (150:150) | 24753773 | 7.43 | 48 | 98.19 | 94.49 | 23067015 | 93.19 | 98.85 | 95.62 |
| C43 | 350 | (150:150) | 24142468 | 7.24 | 49 | 98.09 | 94.28 | 22048126 | 91.33 | 98.82 | 95.52 |
| C51 | 350 | (150:150) | 25277050 | 7.58 | 48 | 98.31 | 94.73 | 23306879 | 92.21 | 98.97 | 95.91 |
| C52 | 350 | (150:150) | 24809988 | 7.44 | 48 | 98.02 | 94.17 | 23328128 | 94.03 | 98.8 | 95.48 |
| C53 | 350 | (150:150) | 25020746 | 7.51 | 48 | 98.22 | 94.53 | 23576905 | 94.23 | 98.86 | 95.62 |
| XH1 | 350 | (150:150) | 25357779 | 7.61 | 55 | 97.9 | 94.1 | 19499083 | 76.9 | 98.9 | 95.82 |
| XH2 | 350 | (150:150) | 25056188 | 7.52 | 55 | 97.8 | 93.87 | 19440470 | 77.59 | 98.82 | 95.58 |
| XH3 | 350 | (150:150) | 24497372 | 7.35 | 42 | 97.24 | 92.95 | 5132230 | 20.95 | 98.84 | 95.66 |
