# Supplementary material for: Changes in microbial community succession and volatile compounds during the natural fermentation of bangcai
Source: Front Microbiol. 2025 Apr 2;16:1581378. doi: 10.3389/fmicb.2025.1581378 (PMC11999953; doi:10.3389/fmicb.2025.1581378)
Supplement: Supplementary file 2 [file Table_2.docx]

**Appendix B. Supplementary Chart**


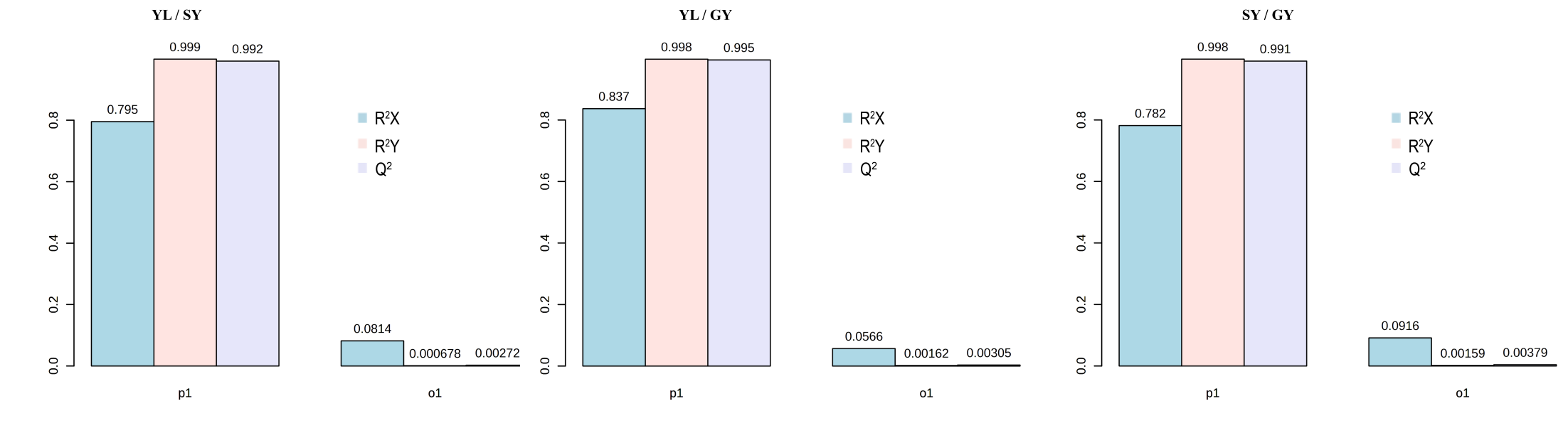


**Fig. S1** Results of OPLS-DA cross-validation testing for three group comparisons: YL/SY, YL/GY, and SY/GY.

Note: The bars represent the model performance metrics: R²X (blue) indicates the proportion of variance in X explained by the model, R²Y (pink) represents the proportion of variance in Y explained, and Q² (purple) reflects the model's predictive power. p1 represents the predictive component, while o1 represents the orthogonal component, which accounts for noise or irrelevant variation. The high R²Y and Q² values across all comparisons demonstrate strong discrimination and predictive performance of the models.


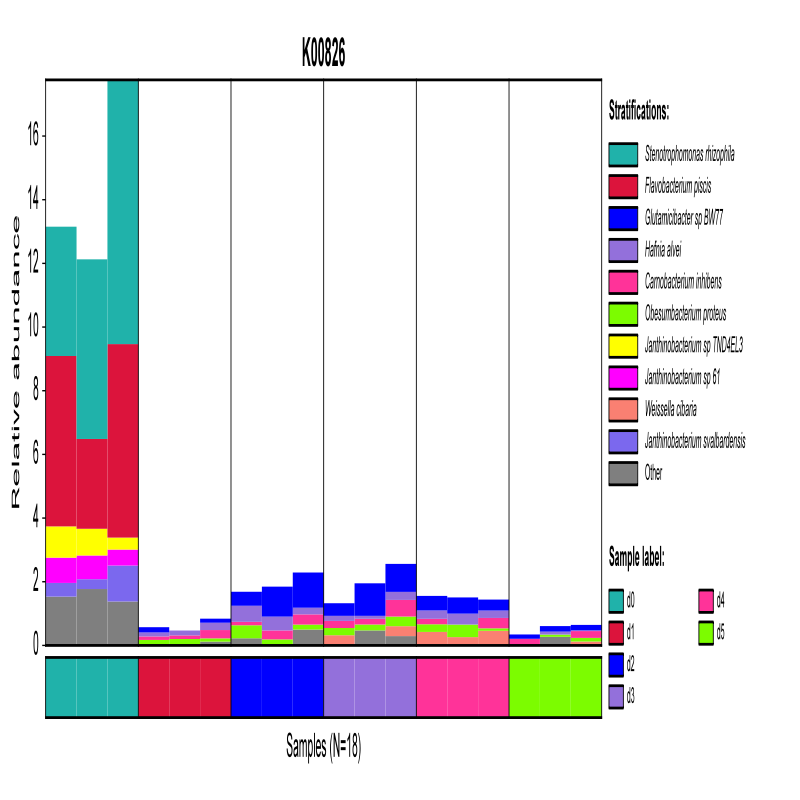


Fig.S2. The relative abundance of K00826 (branched-chain amino acid aminotransferase, BCAT) across sample groups and the contribution analysis of dominant species.
